# Supplementary material for: Mechanism of APTX nicked DNA sensing and pleiotropic inactivation in neurodegenerative disease
Source: EMBO J. 2018 Jun 22;37(14):e98875. doi: 10.15252/embj.201798875 (PMC6043908; doi:10.15252/embj.201798875)
Supplement: Supplementary file 1 — Appendix [file EMBJ-37-e98875-s001.pdf]

Appendix Supplementary Information

Mechanism of APTX Nicked DNA Sensing and Pleiotropic Inactivation in Neurodegenerative Disease

Percy Tumbale[1#], Matthew J. Schellenberg[1#], Geoffrey A. Mueller[1#], Emma Fairweather[2], Mandy Watson[2], Jessica N. Little[1], Juno Krahn[1], Ian Waddell[2], Robert E London[1], and R. Scott Williams[1]*

[1]Genome Integrity and Structural Biology Laboratory, National Institute of Environmental Health Sciences, US National Institutes of Health, Department of Health and Human Services, Research Triangle Park, NC, USA

[2] Drug Discovery Group Cancer Research UK Manchester Institute, Manchester, M20 4BX.

[#]These authors contributed equally to this work

*Correspondence: williamsrs@niehs.nih.gov

Table of Contents:

Appendix Figures S1-S8
Appendix Tables S1-S3

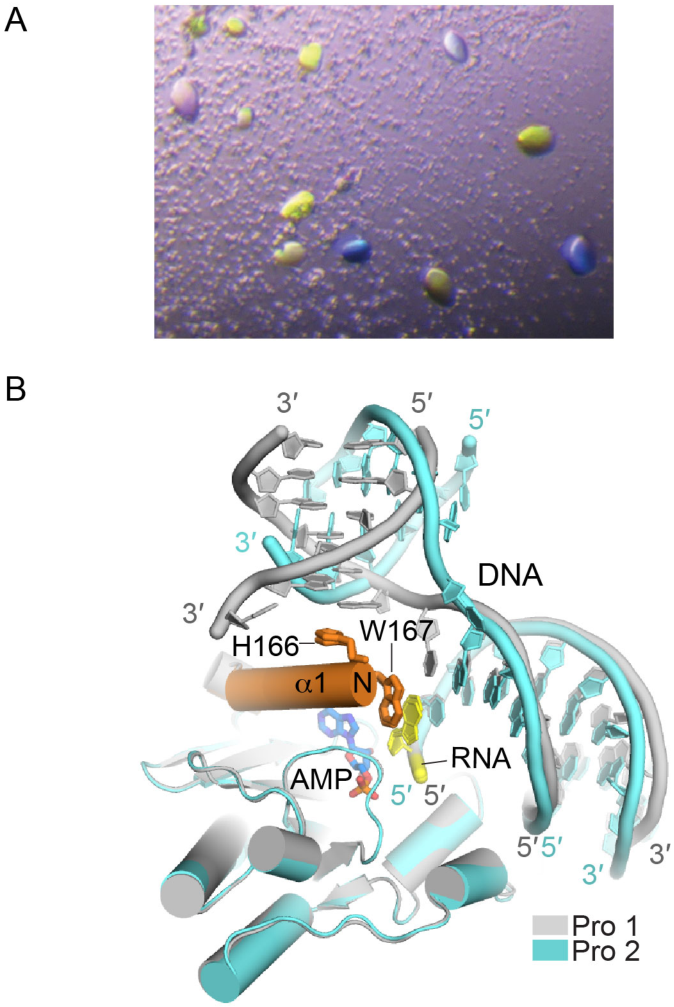

Appendix Figure S1. Crystals of hAPTX-nicked RNA-DNA complex and structural overlay of APTX protomers in an asymmetric unit. (A) Crystals of hAPTX-nicked RNA-DNA complex. **(B)** Structural overlay of Pro 1 and Pro 2 illustrates the two nick-bound APTX molecules bind the upstream region of the nick with different conformations, but both complexes display common features: 1) Cooperative DNA damage sensing by HIT and Znf domains, 2) N-term α1 helix wedging (H166 and W167) into DNA base stack, and 3) DNA bending.

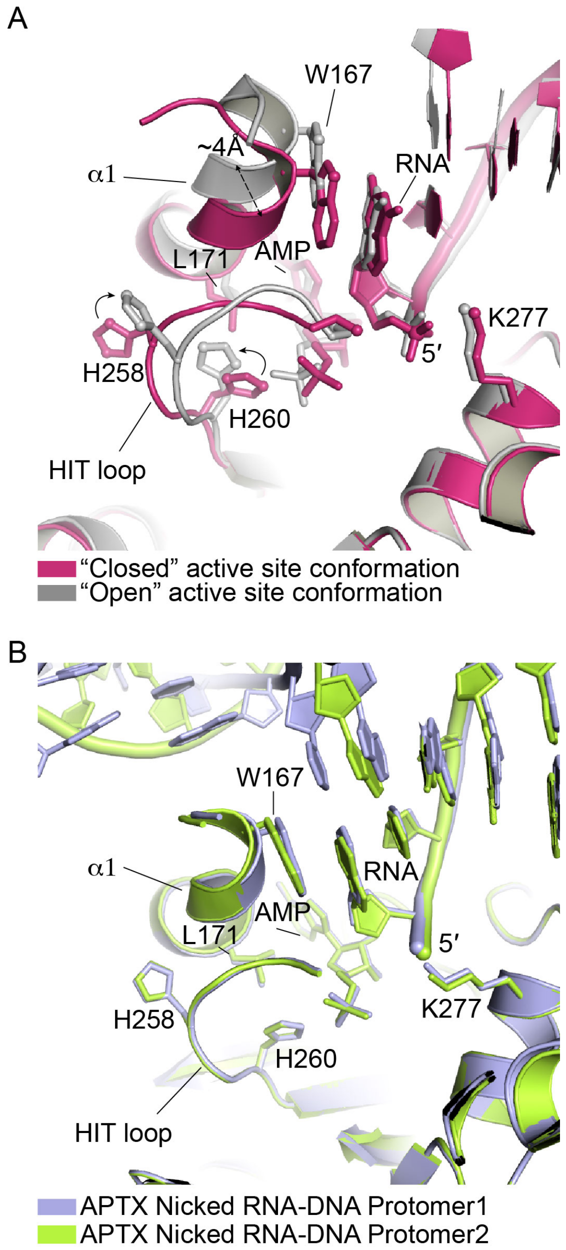

Appendix Figure S2. Active site conformations in APTX structures. (A) X-ray structures of APTX bound to blunt-ended DNA display "open" and "closed" APTX conformations differing by conformational changes in helix α1, and rearrangements of the HIT-loop. Structures are from conformations in APTX-DNA complexes, RCSB code 4NDF). **(B)** Structual superpositions of APTX nicked RNA-DNA protomers 1 and 2 illustrate that the closed assembled active site conformation is found in both of these complexes.

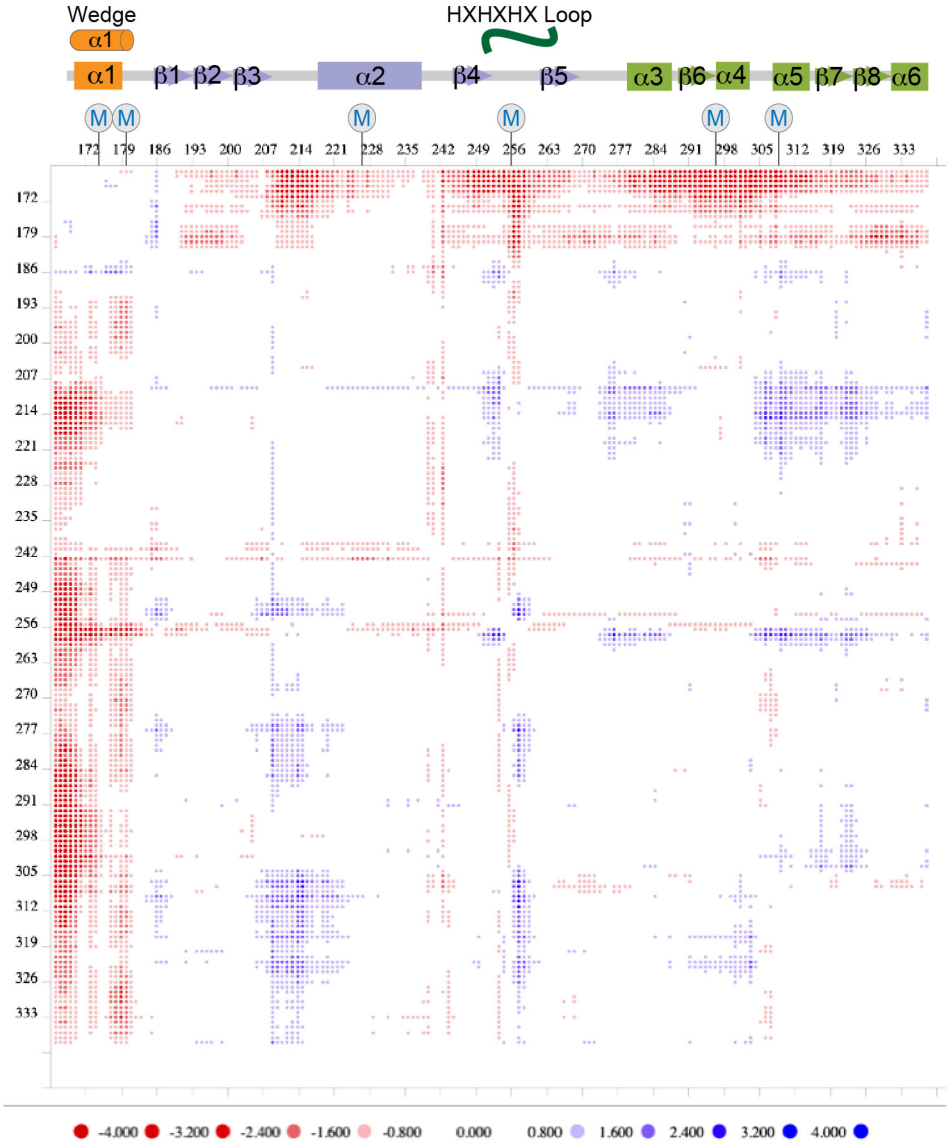

Appendix Figure S3. Distance difference matrix plot (DDMP) of APTX. APTX conformational changes observed in open and closed APTX conformational states (4NDF). Methionine residues labeled in NMR experiments are shown as circled "M"s, and sample the APTX catalytic domain regions that change in crystal structures. Methionine probes and NMR experiments suggest that solution conformational changes monitored in ^{13}C-Met labeled solution samples are coincident with conformational changes observed in APTX X-ray structures.

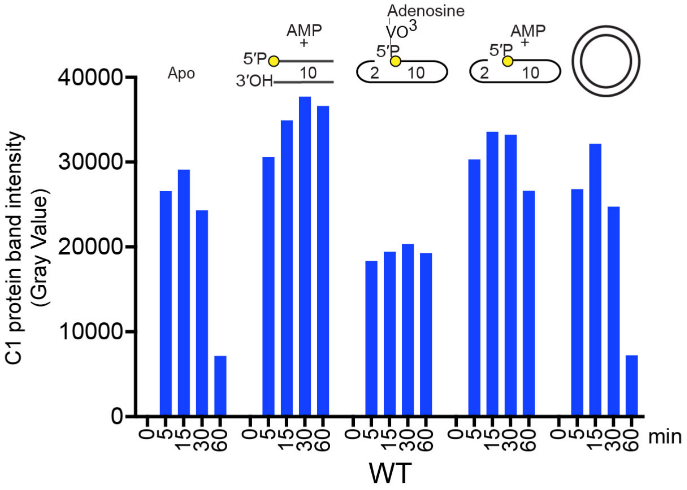

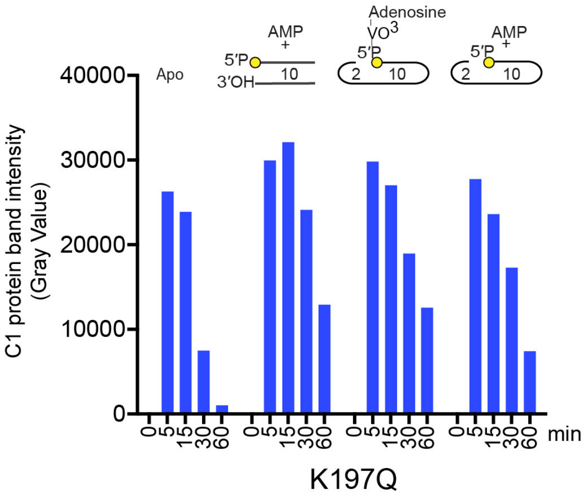

Appendix Figure S4. Proteolytic processing of the C1 fragment. Quantification of "C1" APTX protein fragments generated from chymotryptic proteolysis of APTX wild-type and a DNA binding defective variant K197Q in Apo and ligand bound (blunt-ended DNA, nicked DNA, transition mimic DNA, adenosine and orthovandate $–VO4^{3-}$, or relaxed circular plasmid DNA) conditions. Intensity of C1 protein bands from Figure 2B were measured by ImageJ and plotted as function of time.

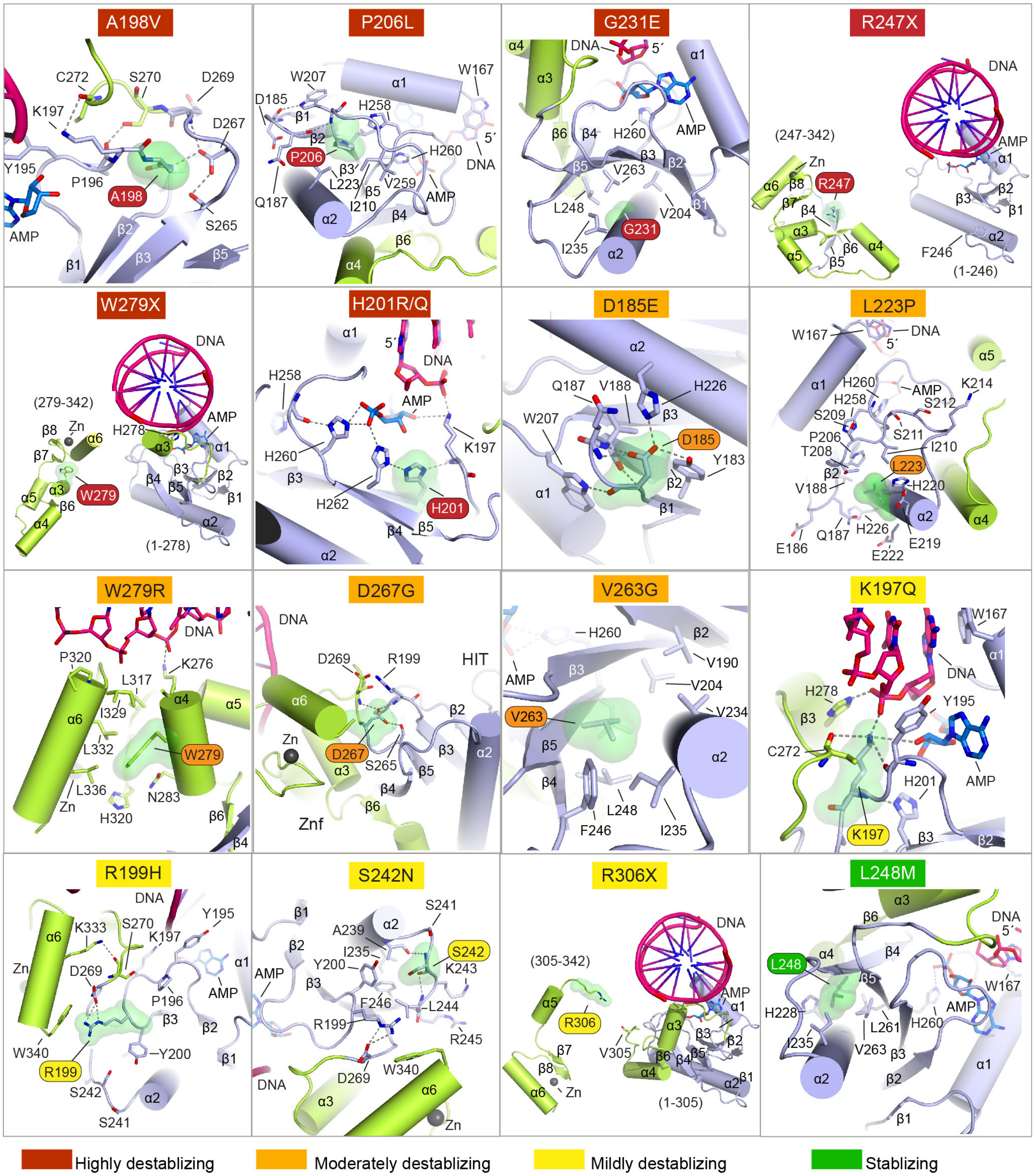

Appendix Figure S5. Molecular environments of APTX residues mutated in AOA1. The mutation labels are colored according to the mutant protein stability classes: 1) highly destabilizing (red), 2) moderately destabilizing (orange), 3) mildly destabilizing (yellow), and 3) stabilizing (green). Mutated residues are highlighted with a green transparent surface. The HIT domain is in blue, Znf domain in green, DNA in pink, AMP in blue. **A198V**: Ala198 maps to a flexible loop flanked by β1 and β2 and is involved in a hydrogen bonding network at the HIT-Znf domain interface. Substitution of a larger valine sidechain would result in steric clash and disruption of domain-domain contacts. **P206L**: Pro206 maps to the carboxyl end of β3, and is involved in protein hydrophobic core Van der Waals interactions. Substitution with a leucine sidechain would create several steric clashes in the protein core. **G231E**: Gly231 located on α2 participates in

Van der Waals interactions involving residues of β3, β4, and β5. Substitution of a larger and charged glutamate introduces a charged sidechain into the protein hydrophobic core. **R247X**: Truncation at Arg247 results in the loss of the entire Znf domain. **W279X**: Truncation at Trp279 results in the loss of the entire Znf domain. **H201R/Q**: His201 is an active site residue participating in the APTX catalytic chemistry. Substitution of H201 with an arginine or glutamine sidechain would distort active site architecture and chemistry. **D185E**: Asp185 participates in a hydrogen-bonding network involving Tyr183 (β1), His226 (α2), and Trp207 (β3-α2 loop). Substitution of a larger glutamate sidechain would likely create steric clashes and distortion of the H-bonding network. **L223P**: Leu223 is located near the junction of a surface loop (β3-α2) and the N-terminal end of α2. Substitution of Leu223 with a proline would disrupt α2, possibly altering the helix trajectory and protein folding. **W279R**: Trp279 located on α4 participates in a hydrophobic core involving stabilizing α6 and the Zn^{2+} binding motif. This mutation introduces a charged residue in the protein hydrophobic core. **D267G**: Asp267 participates in a hydrogen bonding network at the HIT/Znf domain junction, and mutation to glycine would result in loss of many interdomain stabilizing interactions. **V263G**: Val263 is located on β5 and is a hydrophobic core residue A glycine substitution creates a cavity in the protein core. **K197Q**: Lys197 is located on a DNA binding loop opposite to the HxHxH (HIT) loop. The Lys197 sidechain contacts the peptide backbones of Tyr195 and Cys272 (Znf), the phosphate backbone of the continuous DNA strand and the 2′OH of the ribose of AMP. Substitution with glutamine sidechain results in loss of contacts with the DNA substrate, impacting adenylated substrate binding. **R199H**: Arg199 participates in a salt bridge with Asp269 and a cation-π interaction with Trp340. Substitution of a histidine sidechain results in loss of these contacts, altering protein surface and destabilization of the protein. **S242N**: Ser242 located on a surface loop and makes peptide backbone contacts with Leu244 and Ala239. Substitution of asparagine causes rearrangements of these sidechain contacts, resulting in altered surface loop structure. **R306X**: Truncation at Arg306 results in partial loss of the Znf domain. **L248M**: Leu248 located on β4 participates in protein core Van der Waals interactions involving residues of α2 and β5. Substitution of a methionine sidechain results in perturbation of the hydrophobic network, that impact conformational changes during catalytic cycles.

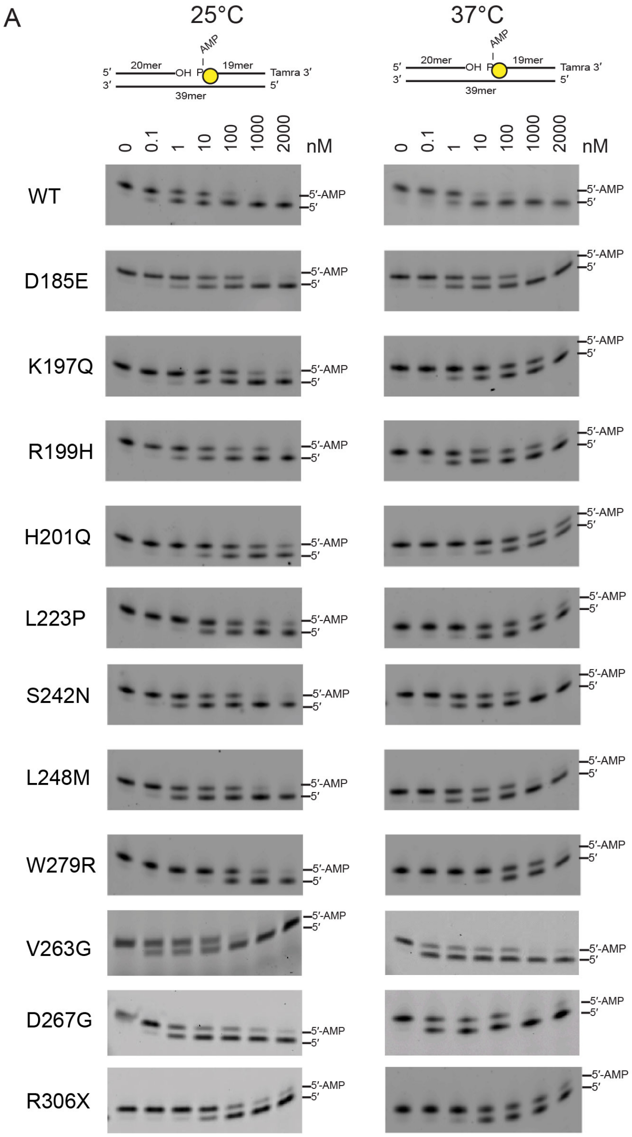

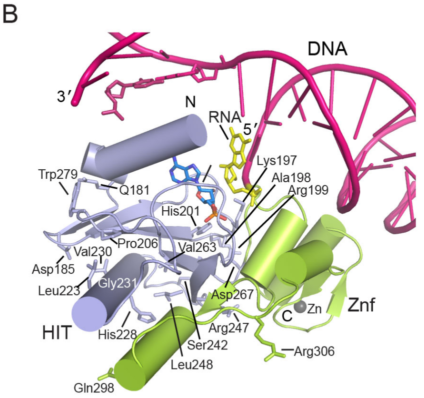

C

APTX	Difference in T_M °C between WT and AOA1 mutants		Fold increase in protein to reach 50% processing of 10nM AMPRNA/DNA	
	APO	AMP	25°C	37°C
WT	0.00	0.00	1.0	1.0
L248M	-0.10	1.23	4.3	6.9
K197Q	-2.71	-5.45	50.8	123.9
S242N	-3.57	-1.70	4.2	8.4
R306X	-3.82	-5.62	11.0	40
R199H	-6.71	-4.57	17.9	13.8
W279R	-7.41	-7.44	2.3	10.3
D267G	-15.56	-15.60	3.1	14.2
D185E	x	-13.89	14.2	15.3
H201Q	x	-18.28	86	694.3
V263G	x	-15.87	4.2	30.4
L223P	x	-13.29	48.6	112.6

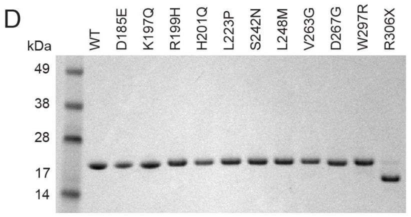

Appendix Figure S6. Effect of AOA1 mutations on APTX activity. (A) Ten-fold dilutions of APTX mutant proteins were tested for deadenylation activity on a 3´-TAMRA labelled 5´-adenylated nicked RNA-DNA duplex substrate at 25ºC and 37ºC. The reaction products were resolved in TBE-urea denaturing gels and visualized by fluorescent scanning. Fold increase of protein to reach 50% activity relative to the wild type hAPTX was displayed. Representative experiment from 3 replicates is shown. **(B)** The positions of APTX mutations found in AOA1 are mapped onto the structure of hAPTX **(C)** A table displaying the difference in T_m values for AOA1 mutants and wild-type hAPTX in unliganded and AMP bound conditions, and AOA1 mutagenic effects on APTX deadenylation activity at 25 ºC and 37 ºC. **(D)** Coomassie blue stained SDS-PAGE showing purified APTX mutant proteins.

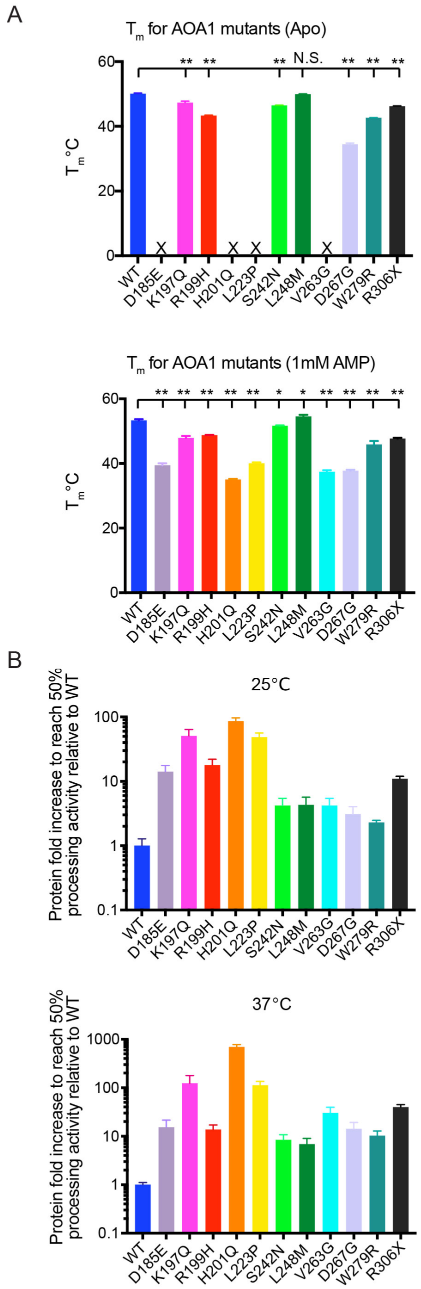

Appendix Figure S7. AOA1 mutagenic effects on APTX thermal stability and catalytic activity. (A) The midpoint temperatures (T_m) for APTX wild-type and mutants in unliganded (top) and AMP bound (bottom) conditions were determined by ThermoFluor assays and displayed in an increasing amino acid number order following the wild-type APTX. (X=unable to determine T_m, * P-value < 0.05, ** P-value < 0.01, N.S.=not-significant. Mean ± s.d. (3 technical replicates) is shown. **(B)** Deadenylation activity of APTX mutants. Ten-fold dilutions of APTX mutant proteins were tested for deadenylation on a 3′-TAMRA-labeled 5′-adenylated nicked RNA-DNA substrate at 25 °C (top) and 37 °C (bottom). Fold increase of protein to reach 50 % activity relative to wild-type APTX is displayed in an increasing amino acid number order following the WT protein. Mean ± s.d. (3 technical replicates) is shown.

Overlays of ^{1}H-^{15}N HSQC spectra of
APTX-WT unliganded vs. L248M unliganded

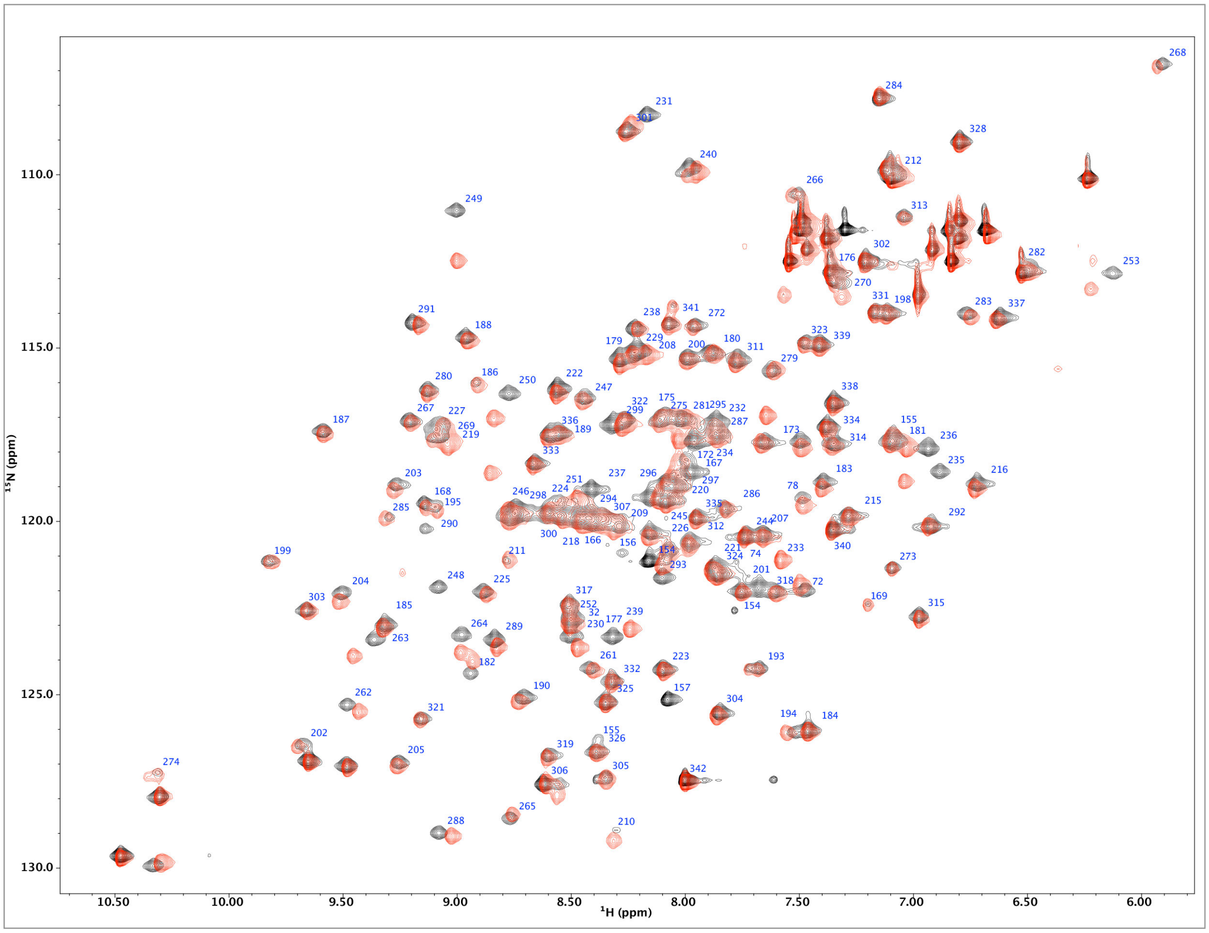

Appendix Figure S8. NMR analysis of L248M. Overlays of assigned ^{1}H-^{15}N HSQC NMR spectra attributed to the wild-type APTX unliganded (black) and L248M unliganded (red).

Appendix Table S1. Locations of the [methyl-13C] labeled methionine residues and their distances relative to the active site (AMP phosphorous) and N-terminal α1 helix-DNA contact (W167Cα).

$^{13}CH_3$ Labaled Met	Location	Distance from MetCε to AMP phosporous	Distance from MetCε to W167Cα	Residue characteristic
Met164	α1	17.9Å	8.6Å	Non-native residue left behind from His tag cleavge
Met175	α1	14.9Å	13.9Å	Hydrophobic core residue
Met180	α1	17.7Å	16.8Å	Solvent expossed residue
Met256	HIT-loop	5.3Å	7.9Å	Activity site residue
Met227	α2	16.2Å	23.7Å	Hydrophobic core residue
Met296	α4	20.8Å	30.7Å	Hydrophobic core residue
Met309	α5	13.8Å	22.9Å	Hydrophobic core residue

Appendix Table S2. Data collection and refinement statistics (molecular replacement)

	APTX nick	R199H	H201Q	S242N	L248M	V263G
PDB code	6CVO	6CVP	6CVQ	6CVR	6CVS	6CVT
**Data collection**						
Space group	$P2_12_12_1$	$P2_12_12_1$	$P2_12_12_1$	$P2_12_12_1$	$P2_12_12_1$	$P2_12_12_1$
Cell dimensions						
a, *b*, *c* (Å)	58.91, 72.55, 147.16	39.51, 121.10, 122.31	40.57, 116.05, 117.22	40.61, 116.06, 117.56	40.38, 116.32, 117.56	40.73, 113.13, 124.50
α, β, γ (°)	90, 90, 90	90, 90, 90	90, 90, 90	90, 90, 90	90, 90, 90	90, 90, 90
Resolution (Å)	50-2.40 (2.49-2.40)	50-2.00 (2.07-2.00)	50-1.65 (1.71-1.65)	50-1.88 (1.95-1.88)	50-2.11 (2.19-2.11)	50-2.94 (3.05-2.94)
R_{sym} or R_{merge}	0.145 (0.571)	0.049 (0.475)	0.076 (0.542)	0.078 (0.517)	0.106 (0.583)	0.071 (0.406)
$I / \sigma I$	11.7 (2.4)	20.0 (1.6)	20.6 (2.0)	16.9 (2.4)	13.8 (2.3)	17.1 (2.1)
Completeness (%)	100 (99.9)	97.2 (83.9)	99.4 (94.2)	97.3 (80.3)	95.4 (96.9)	99.4 (96.9)
Redundancy	4.5 (4.5)	3.3 (2.0)	2.8 (4.1)	4.2 (3.9)	4.7 (4.7)	4.0 (3.9)
**Refinement**						
Resolution (Å)	43.7-2.4	27.6-2.0	32.4-1.65	38.4-1.88	28.9-2.11	39.0-2.94
No. reflections	25386	37198	65829	42732	31260	11635
R_{work} / R_{free}	0.180/0.221	0.170/0.218	0.118/0.167	0.167/0.190	0.162/0.204	0.207/0.266
No. atoms						
Protein	4114	3807	3953	3823	3968	3763
Ligand/ion	10	3	23	2	10	2
Water	404	510	759	589	498	11
B-factors						
Protein	33.3	32.2	17.7	28.1	39.2	75.3
Ligand/ion	33.5	30.8	34.9	47.2	49.7	113.9
Water	34.4	40.0	33.7	38.2	46.2	46.3
R.m.s. deviations						
Bond lengths (Å)	0.006	0.008	0.009	0.009	0.007	0.008
Bond angles (°)	0.95	1.27	1.33	1.30	1.05	1.19

Each dataset was collected from a single crystal. *Values in parentheses are for highest-resolution shell.

Appendix Table S3. Synthetic oligonucleotides used in biochemistry and crystallization experiments.

Oligo Number	Oligo Name	5′ modification	Sequence 5′ - 3′	3′ modification
1	10ridown-2up-hairpin	Phosphate, Ribose	(rG)TT CTA GAA CGA AGT TCT AGA AC GC GAA GC	None
2	5Pri-10	Phosphate, Ribose	(rG)TTATGATTC	None
3	5Pri-10C	None	GAATCATAAC	None
4	nick 5Pri16-overlap-6	Phosphate, Ribose	(rG)TTCTATATATAGAACGCTGTT	None
5	nick-6up	None	AACAGC	None
6	5P-10-palindrom	Phosphate	GTTCTAGAAC	None
