# Supplementary material for: Mechanism of APTX nicked DNA sensing and pleiotropic inactivation in neurodegenerative disease
Source: EMBO J. 2018 Jun 22;37(14):e98875. doi: 10.15252/embj.201798875 (PMC6043908; doi:10.15252/embj.201798875)
Supplement: Supplementary file 2 — Dataset EV1 [file EMBJ-37-e98875-s002.pdf]

Tumbale *et al.*, "**Mechanism of APTX Nicked DNA Sensing and Pleiotropic Inactivation in Neurodegenerative Disease**"

Expanded View Dataset EV1:

Thermal Shift Assay Data linked to Figure 3C

Well position	Protein sample	Conditions	Calculated Tm (˚C)	Average Tm (˚C)	SD	Delta Tm (˚C)
A1	WT	DMSO	50.23	50.05	0.2	3.31
A2	WT	DMSO	49.83			
A3	WT	DMSO	50.1			
A4	WT	AMP	53	53.36	0.36	
A5	WT	AMP	53.37			
A6	WT	AMP	53.71			
A7	D185E	DMSO	unstable			-
A8	D185E	DMSO	unstable			
A9	D185E	DMSO	unstable			
A10	D185E	AMP	40.04	39.47	0.6	
A11	D185E	AMP	39.52			
A12	D185E	AMP	38.84			
B1	K197Q	DMSO	47.66	47.34	0.43	0.57
B2	K197Q	DMSO	47.51			
B3	K197Q	DMSO	46.86			
B4	K197Q	AMP	48.57	47.91	0.6	
B5	K197Q	AMP	47.41			
B6	K197Q	AMP	47.75			
B7	R199H	DMSO	43.19	43.22	0.09	5.57
B8	R199H	DMSO	43.32			
B9	R199H	DMSO	43.15			
B10	R199H	AMP	48.79	48.79	0.06	
B11	R199H	AMP	48.73			
B12	R199H	AMP	48.85			
C1	H201Q	DMSO	unstable			-
C2	H201Q	DMSO	unstable			
C3	H201Q	DMSO	unstable			
C4	H201Q	AMP	35.07	35.08	0.18	
C5	H201Q	AMP	34.91			
C6	H201Q	AMP	35.26			
C7	L223P	DMSO	unstable			-
C8	L223P	DMSO	unstable			
C9	L223P	DMSO	unstable			
C10	L223P	AMP	40.09	40.07	0.33	
C11	L223P	AMP	39.74			
C12	L223P	AMP	40.39			
D1	S242N	DMSO	46.51	46.48	0.04	5.19

D2	S242N	DMSO	46.43			
D3	S242N	DMSO	46.49			
D4	S242N	AMP	51.52	51.66	0.18	
D5	S242N	AMP	51.6			
D6	S242N	AMP	51.87			
D7	L248M	DMSO	50	49.95	0.06	4.64
D8	L248M	DMSO	49.97			
D9	L248M	DMSO	49.88			
D10	L248M	AMP	54.35	54.59	0.5	
D11	L248M	AMP	54.25			
D12	L248M	AMP	55.17			
E1	D267G	DMSO	34.18	34.49	0.35	3.27
E2	D267G	DMSO	34.87			
E3	D267G	DMSO	34.43			
E4	D267G	AMP	38.12	37.76	0.33	
E5	D267G	AMP	37.48			
E6	D267G	AMP	37.68			
E7	V263G	DMSO	unstable			-
E8	V263G	DMSO	unstable			
E9	V263G	DMSO	unstable			
E10	V263G	AMP	37.11	37.49	0.42	
E11	V263G	AMP	37.43			
E12	V263G	AMP	37.94			
F1	W297R	DMSO	42.6	42.64	0.05	3.28
F2	W297R	DMSO	42.64			
F3	W297R	DMSO	42.69			
F4	W297R	AMP	45.39	45.92	1.05	
F5	W297R	AMP	45.25			
F6	W297R	AMP	47.13			
F7	R306X	DMSO	46.35	46.23	0.11	1.51
F8	R306X	DMSO	46.19			
F9	R306X	DMSO	46.15			
F10	R306X	AMP	47.76	47.74	0.24	
F11	R306X	AMP	47.5			
F12	R306X	AMP	47.97			

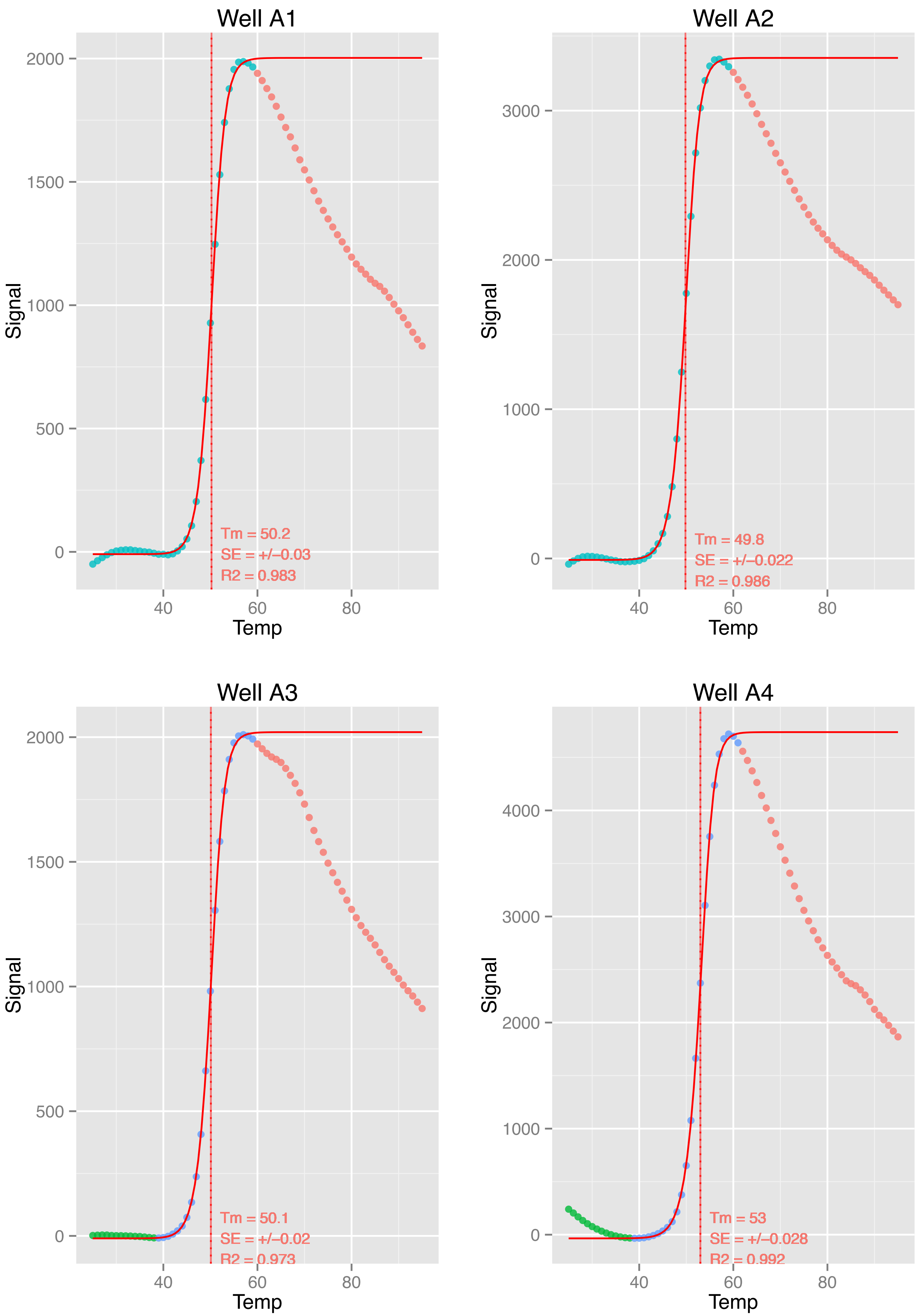

Well A1
Signal
Temp
Tm = 50.2
SE = +/−0.03
R2 = 0.983
Well A2
Signal
Temp
Tm = 49.8
SE = +/−0.022
R2 = 0.986
Well A3
Signal
Temp
Tm = 50.1
SE = +/−0.02
R2 = 0.973
Well A4
Signal
Temp
Tm = 53
SE = +/−0.028
R2 = 0.992

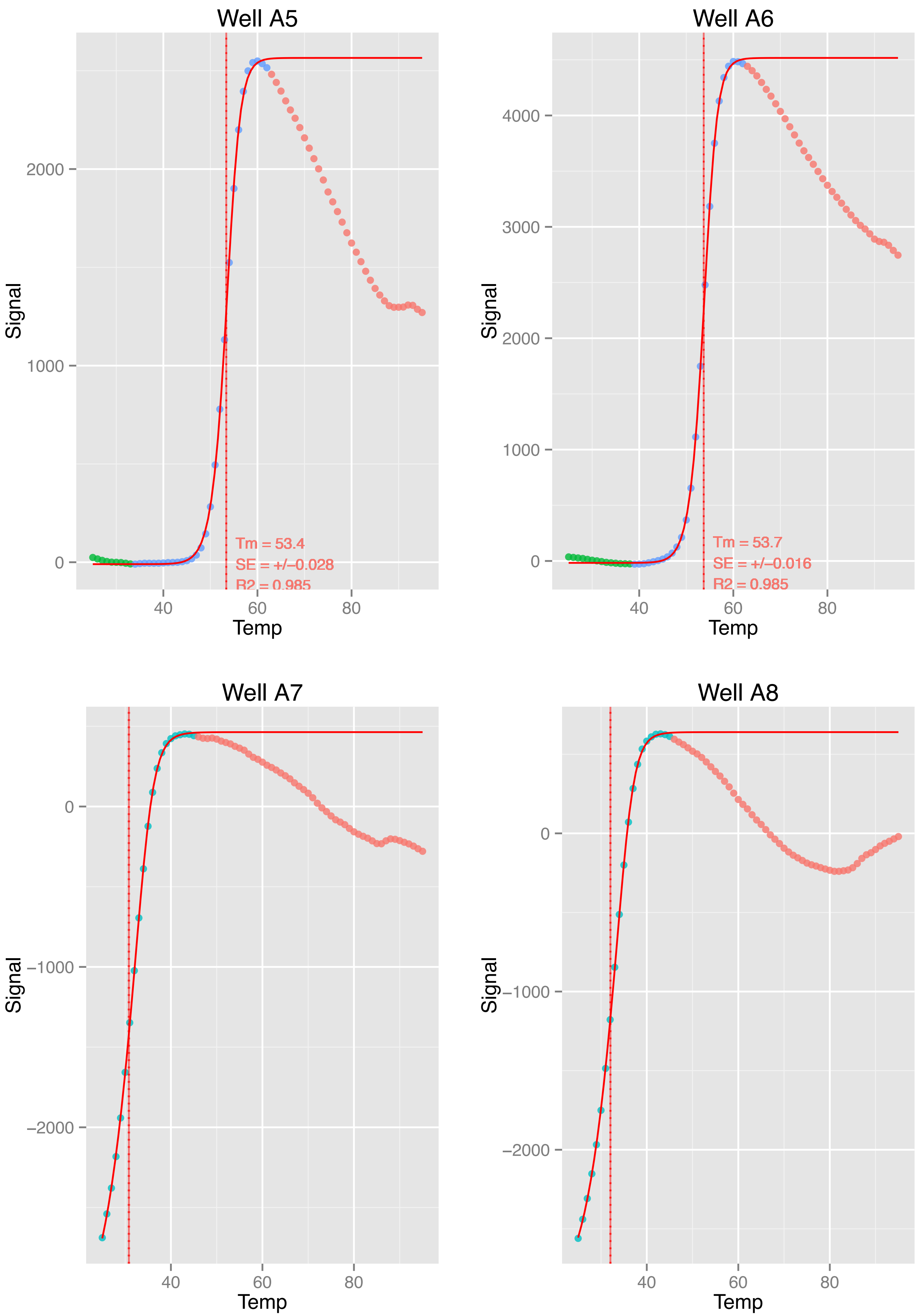
Well A5
Tm = 53.4
SE = +/−0.028
R2 = 0.985
Well A6
Tm = 53.7
SE = +/−0.016
R2 = 0.985
Well A7
Well A8
Signal
Temp

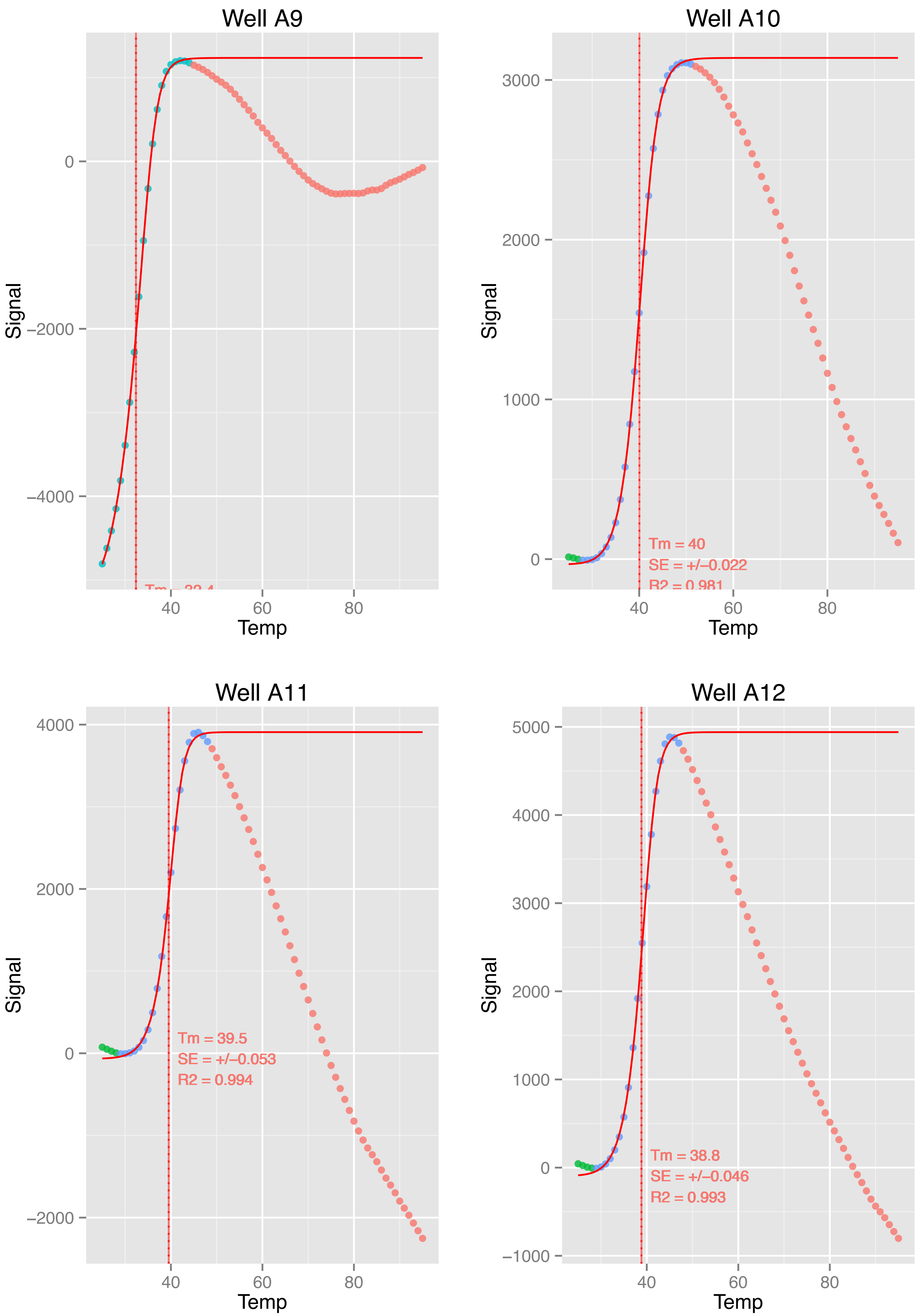

Well A9
Signal
Temp
0
−2000
−4000
40
60
80
Well A10
Signal
Temp
3000
2000
1000
0
40
60
80
Tm = 40
SE = +/−0.022
R2 = 0.981
Well A11
Signal
Temp
4000
2000
0
−2000
40
60
80
Tm = 39.5
SE = +/−0.053
R2 = 0.994
Well A12
Signal
Temp
5000
4000
3000
2000
1000
0
−1000
40
60
80
Tm = 38.8
SE = +/−0.046
R2 = 0.993

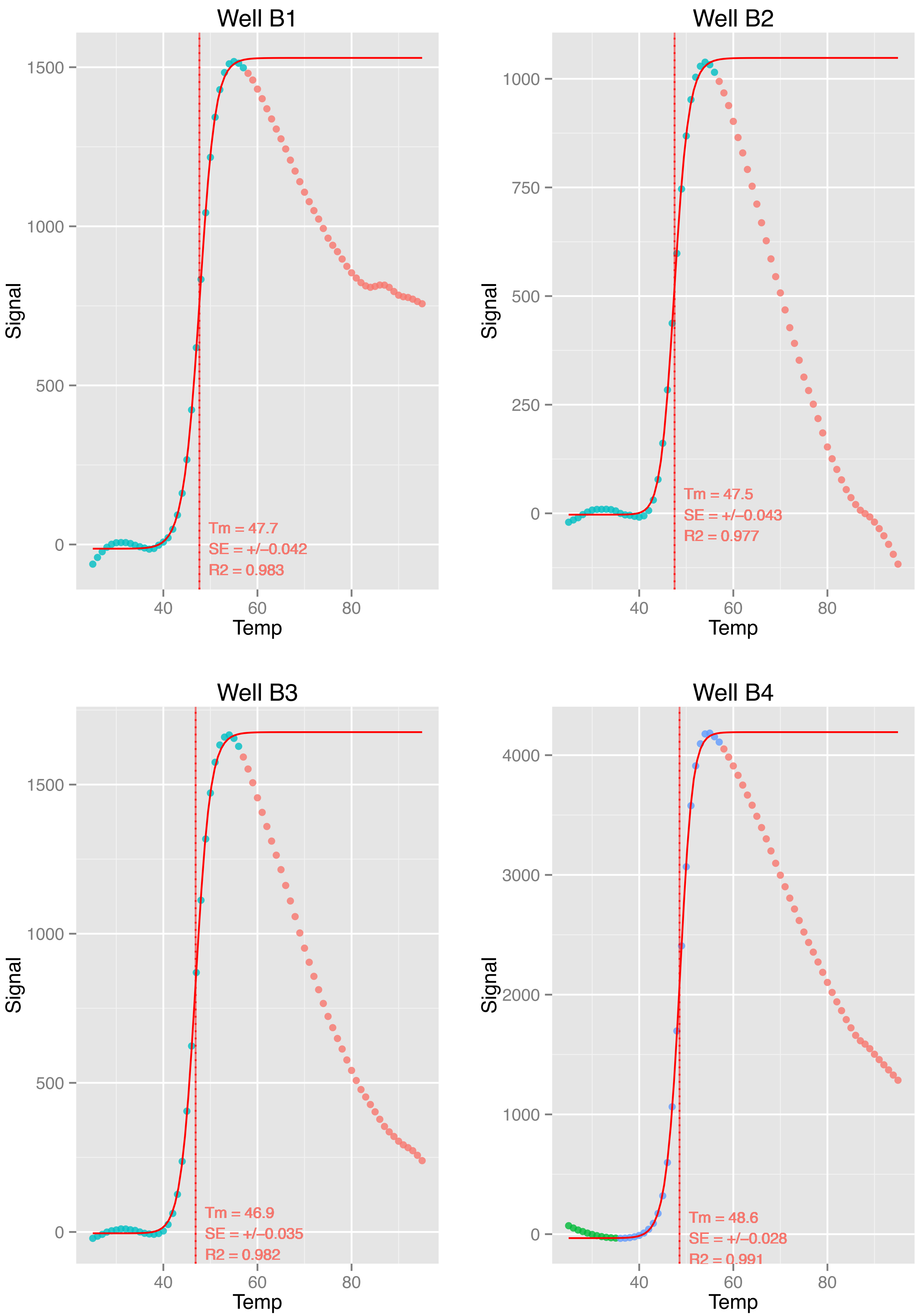
Well B1
Signal
Temp
Tm = 47.7
SE = +/−0.042
R2 = 0.983
Well B2
Signal
Temp
Tm = 47.5
SE = +/−0.043
R2 = 0.977
Well B3
Signal
Temp
Tm = 46.9
SE = +/−0.035
R2 = 0.982
Well B4
Signal
Temp
Tm = 48.6
SE = +/−0.028
R2 = 0.991

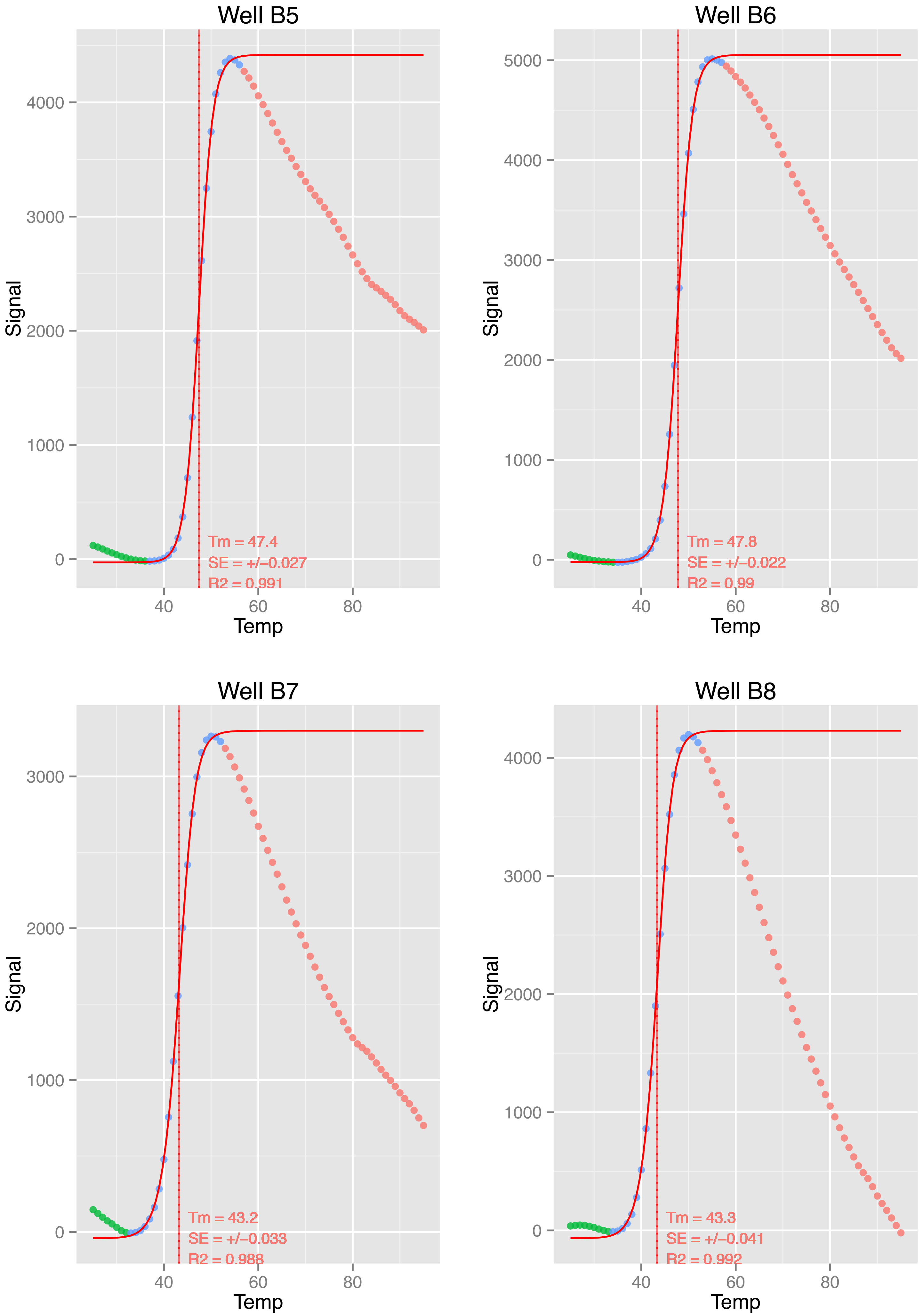
Well B5
Tm = 47.4
SE = +/−0.027
R2 = 0.991
Well B6
Tm = 47.8
SE = +/−0.022
R2 = 0.99
Well B7
Tm = 43.2
SE = +/−0.033
R2 = 0.988
Well B8
Tm = 43.3
SE = +/−0.041
R2 = 0.992
Signal
Temp

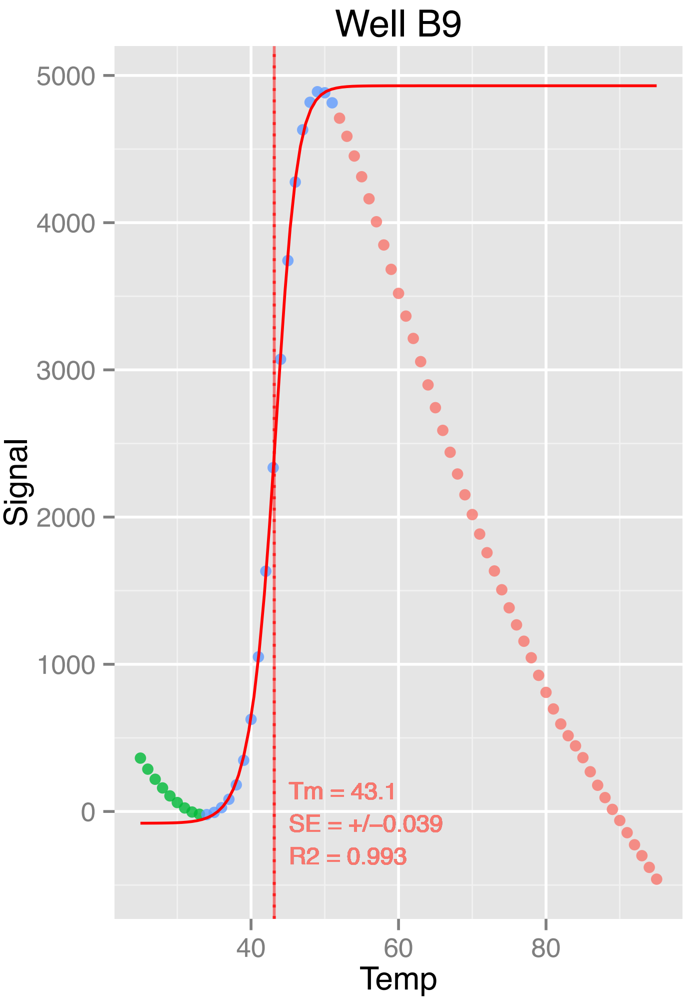
Well B9
Signal
Temp
5000
4000
3000
2000
1000
0
40
60
80
Tm = 43.1
SE = +/−0.039
R2 = 0.993

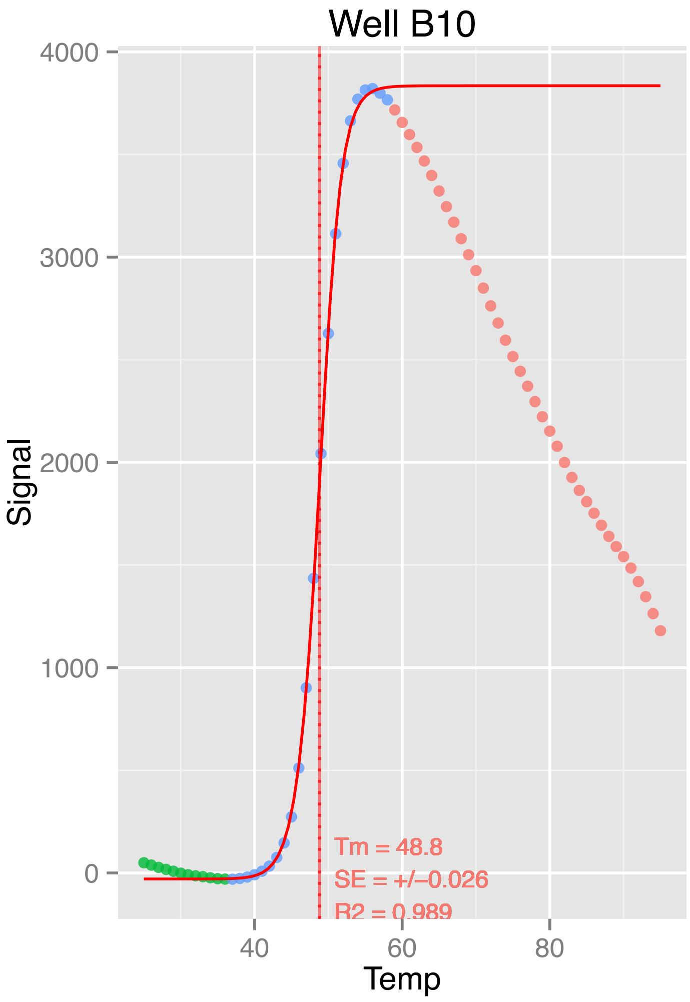
Well B10
Signal
Temp
4000
3000
2000
1000
0
40
60
80
Tm = 48.8
SE = +/−0.026
R2 = 0.989

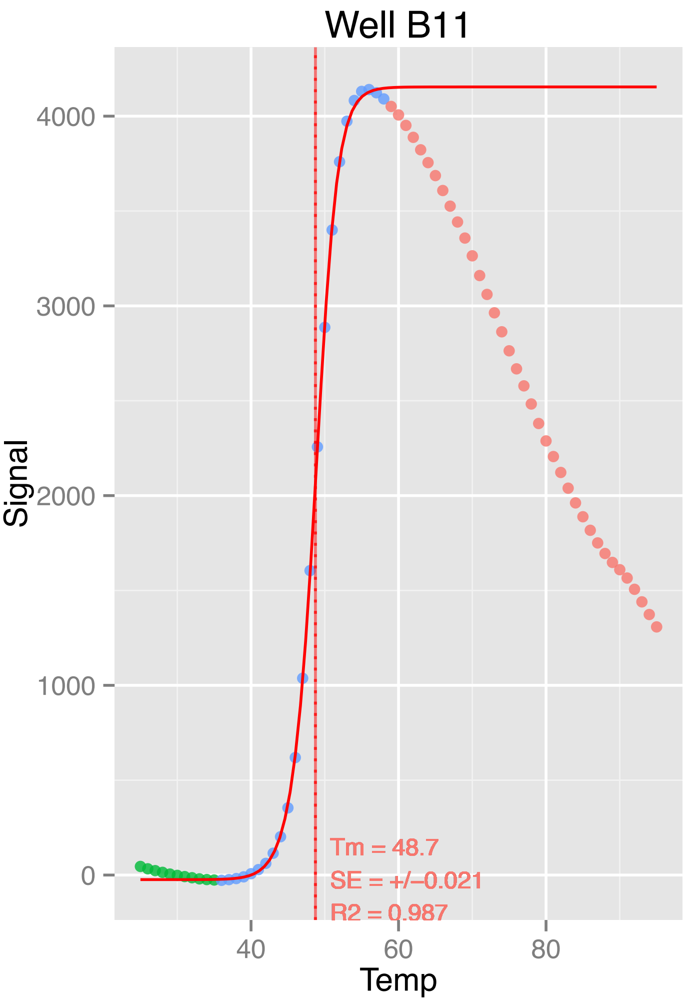
Well B11
Signal
Temp
4000
3000
2000
1000
0
40
60
80
Tm = 48.7
SE = +/−0.021
R2 = 0.987

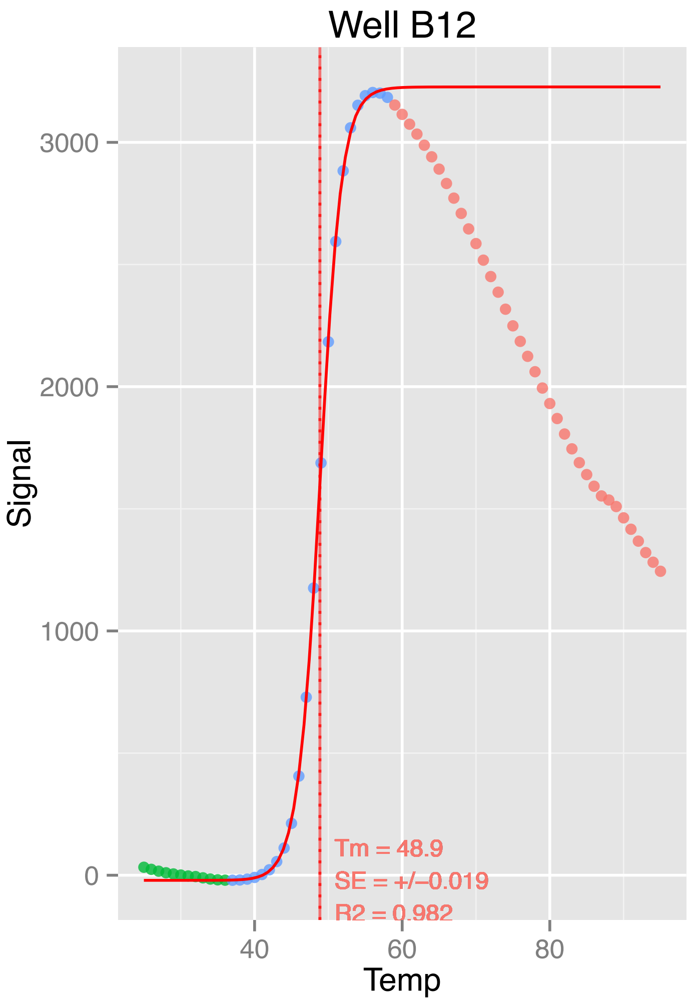
Well B12
Signal
Temp
3000
2000
1000
0
40
60
80
Tm = 48.9
SE = +/−0.019
R2 = 0.982

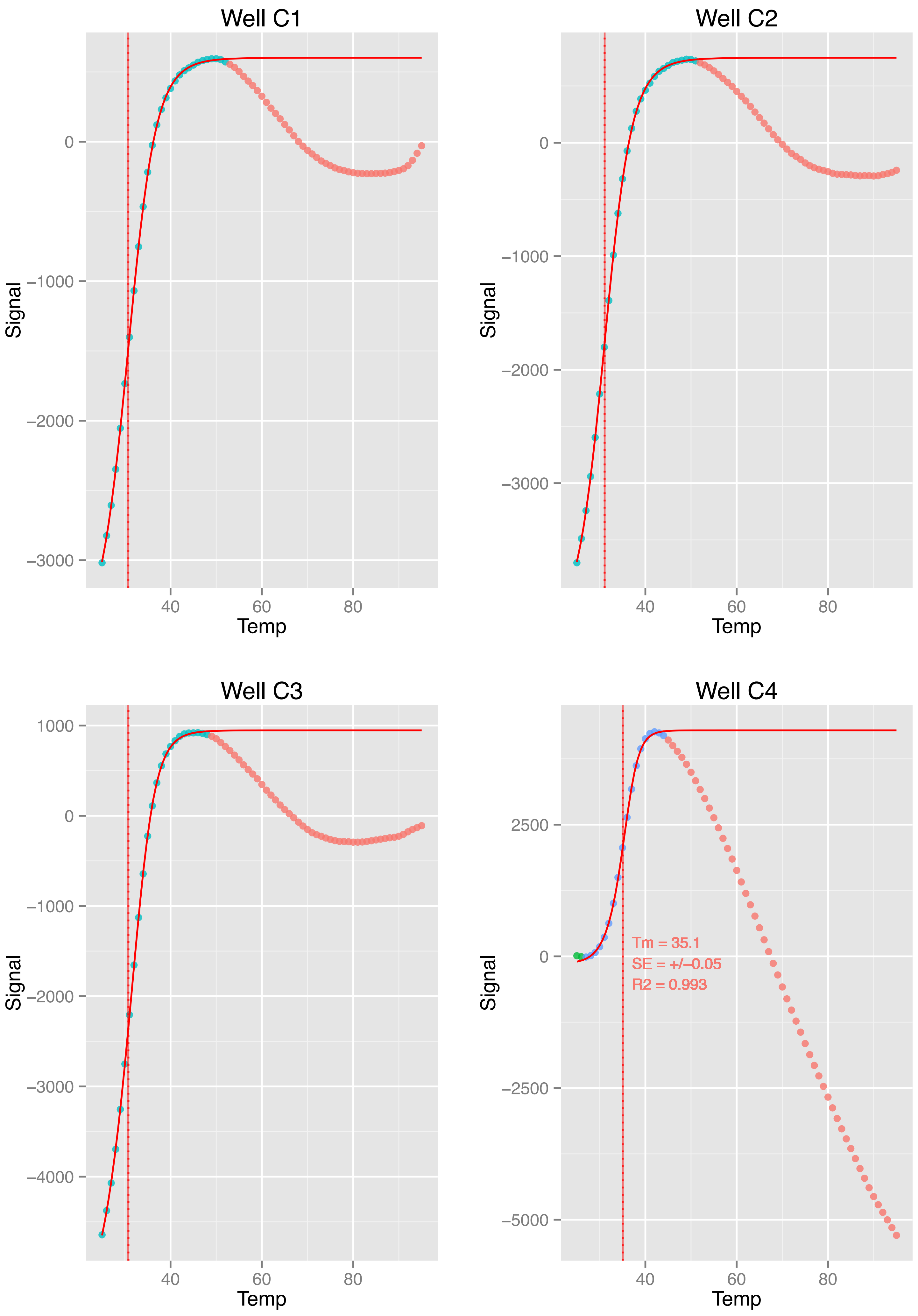
Well C1
Signal
0
−1000
−2000
−3000
40
60
80
Temp
Well C2
Signal
0
−1000
−2000
−3000
40
60
80
Temp
Well C3
Signal
1000
0
−1000
−2000
−3000
−4000
40
60
80
Temp
Well C4
Signal
2500
0
−2500
−5000
40
60
80
Temp
Tm = 35.1
SE = +/−0.05
R2 = 0.993

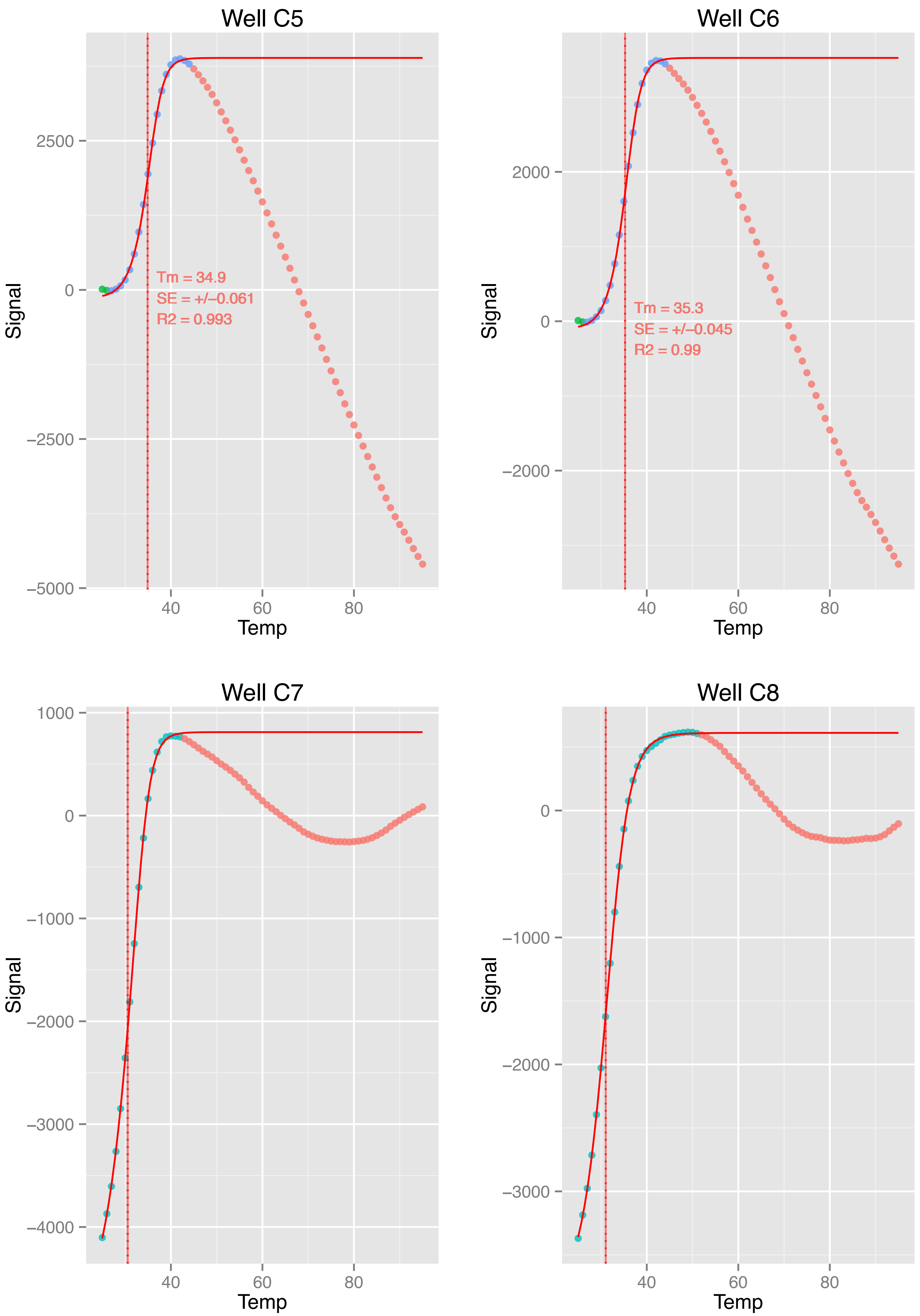

Well C5
Tm = 34.9
SE = +/−0.061
R2 = 0.993
Signal
Temp
2500
0
−2500
−5000
40
60
80
Well C6
Tm = 35.3
SE = +/−0.045
R2 = 0.99
Signal
Temp
2000
0
−2000
40
60
80
Well C7
Signal
Temp
1000
0
−1000
−2000
−3000
−4000
40
60
80
Well C8
Signal
Temp
0
−1000
−2000
−3000
40
60
80

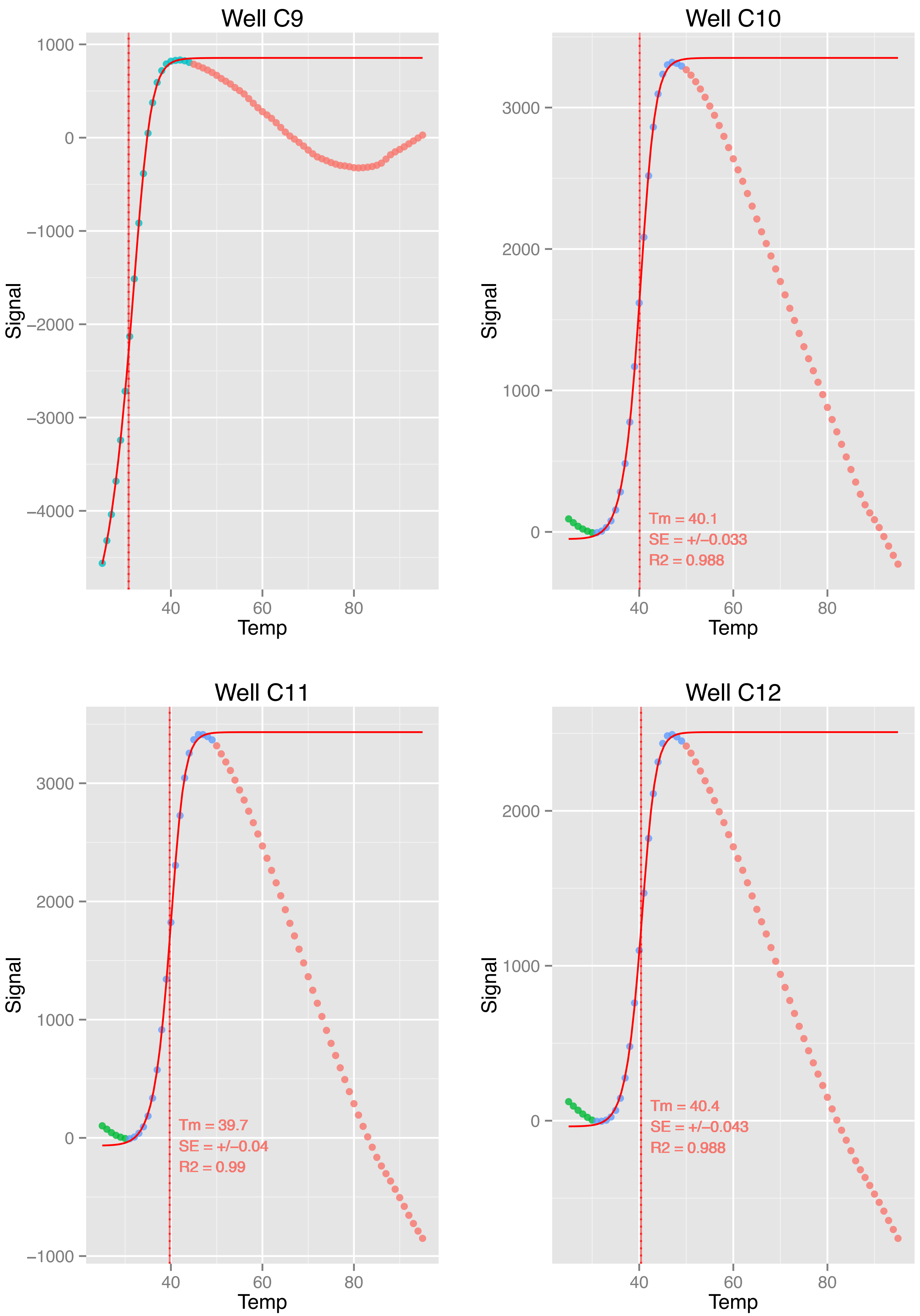
Well C9
Signal
Temp
Well C10
Signal
Temp
Tm = 40.1
SE = +/−0.033
R2 = 0.988
Well C11
Signal
Temp
Tm = 39.7
SE = +/−0.04
R2 = 0.99
Well C12
Signal
Temp
Tm = 40.4
SE = +/−0.043
R2 = 0.988

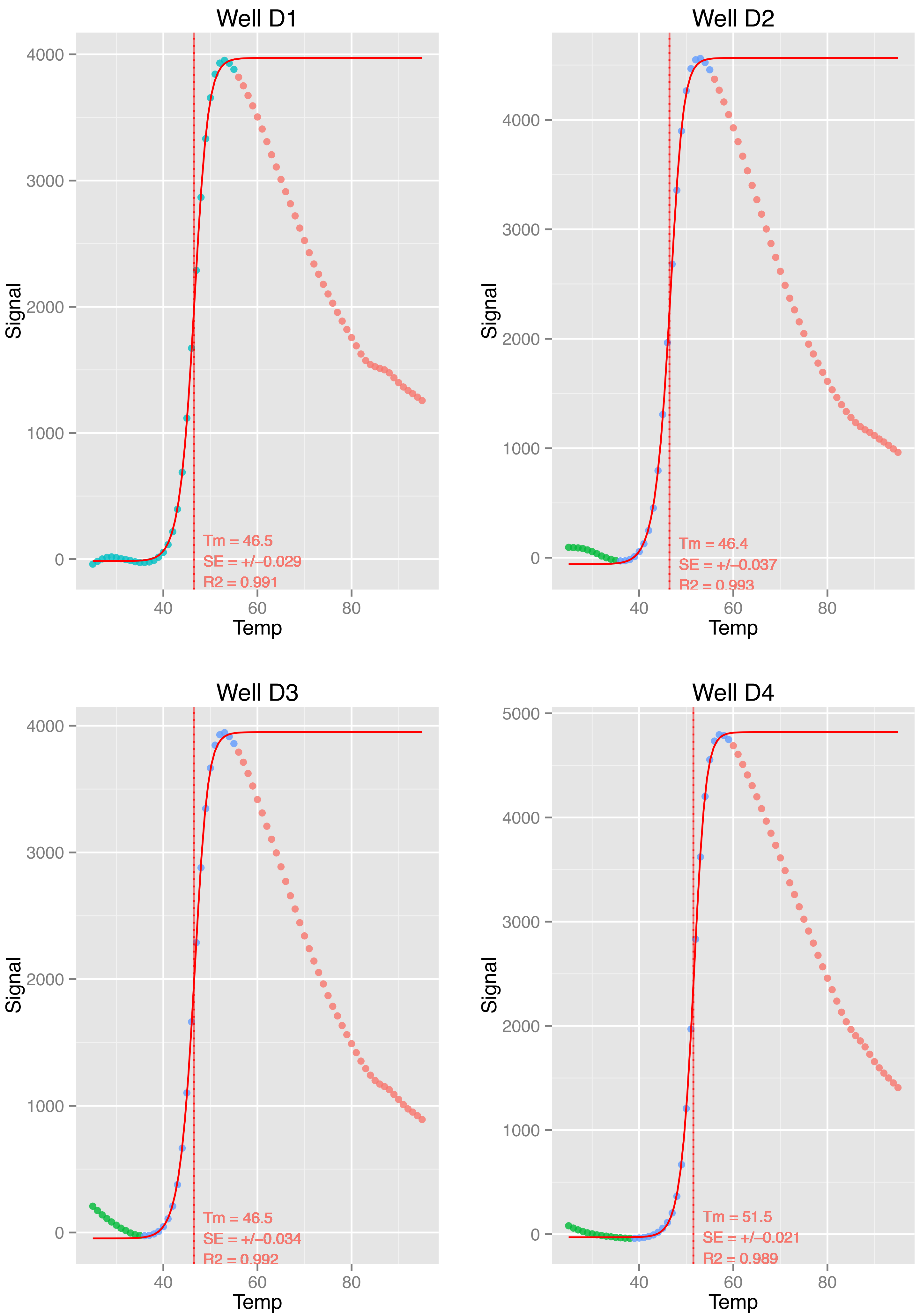
Well D1
Signal
Temp
4000
3000
2000
1000
0
40
60
80
Tm = 46.5
SE = +/−0.029
R2 = 0.991
Well D2
Signal
Temp
4000
3000
2000
1000
0
40
60
80
Tm = 46.4
SE = +/−0.037
R2 = 0.993
Well D3
Signal
Temp
4000
3000
2000
1000
0
40
60
80
Tm = 46.5
SE = +/−0.034
R2 = 0.992
Well D4
Signal
Temp
5000
4000
3000
2000
1000
0
40
60
80
Tm = 51.5
SE = +/−0.021
R2 = 0.989

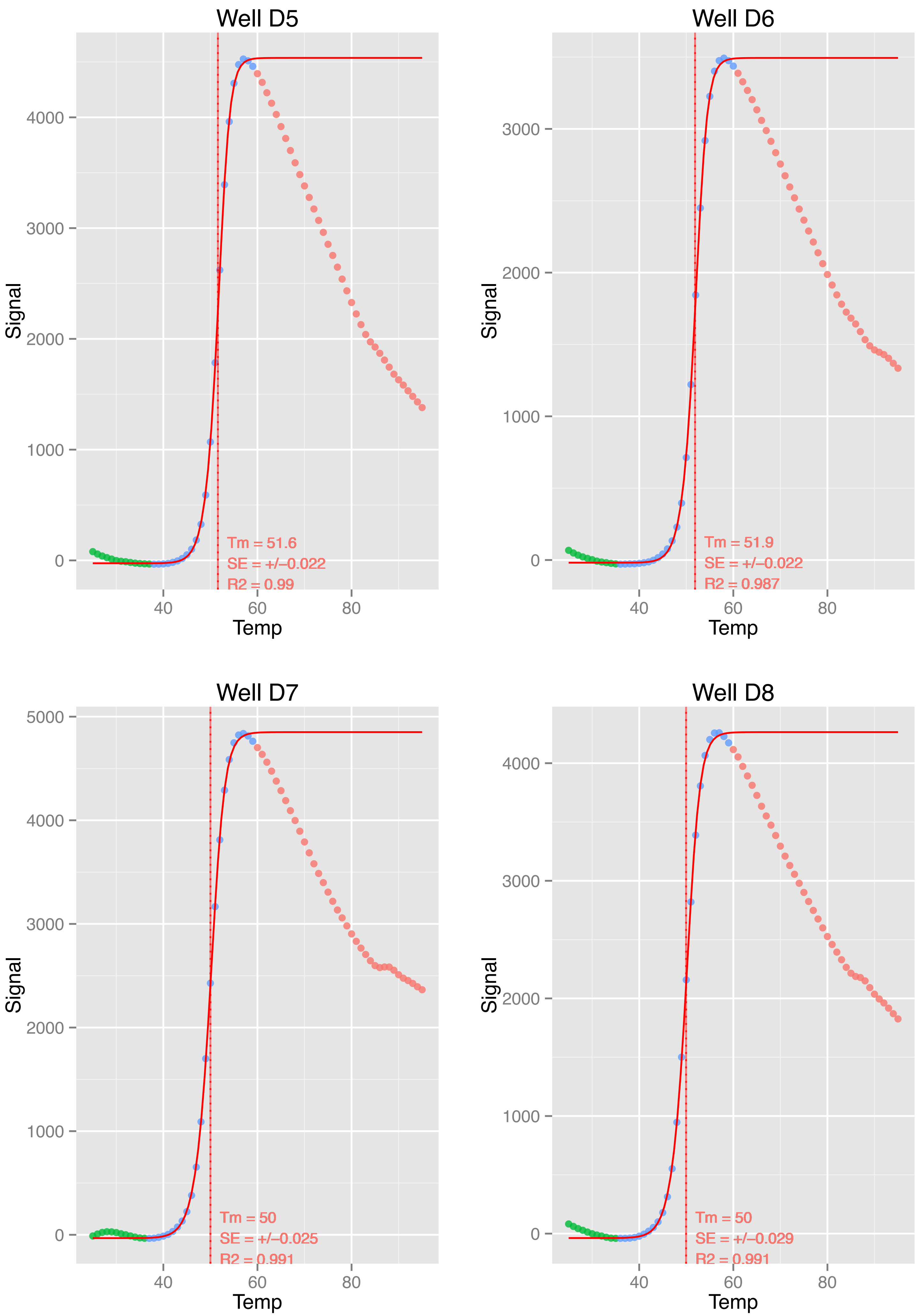
Well D5
Tm = 51.6
SE = +/−0.022
R2 = 0.99
Signal
Temp
Well D6
Tm = 51.9
SE = +/−0.022
R2 = 0.987
Signal
Temp
Well D7
Tm = 50
SE = +/−0.025
R2 = 0.991
Signal
Temp
Well D8
Tm = 50
SE = +/−0.029
R2 = 0.991
Signal
Temp

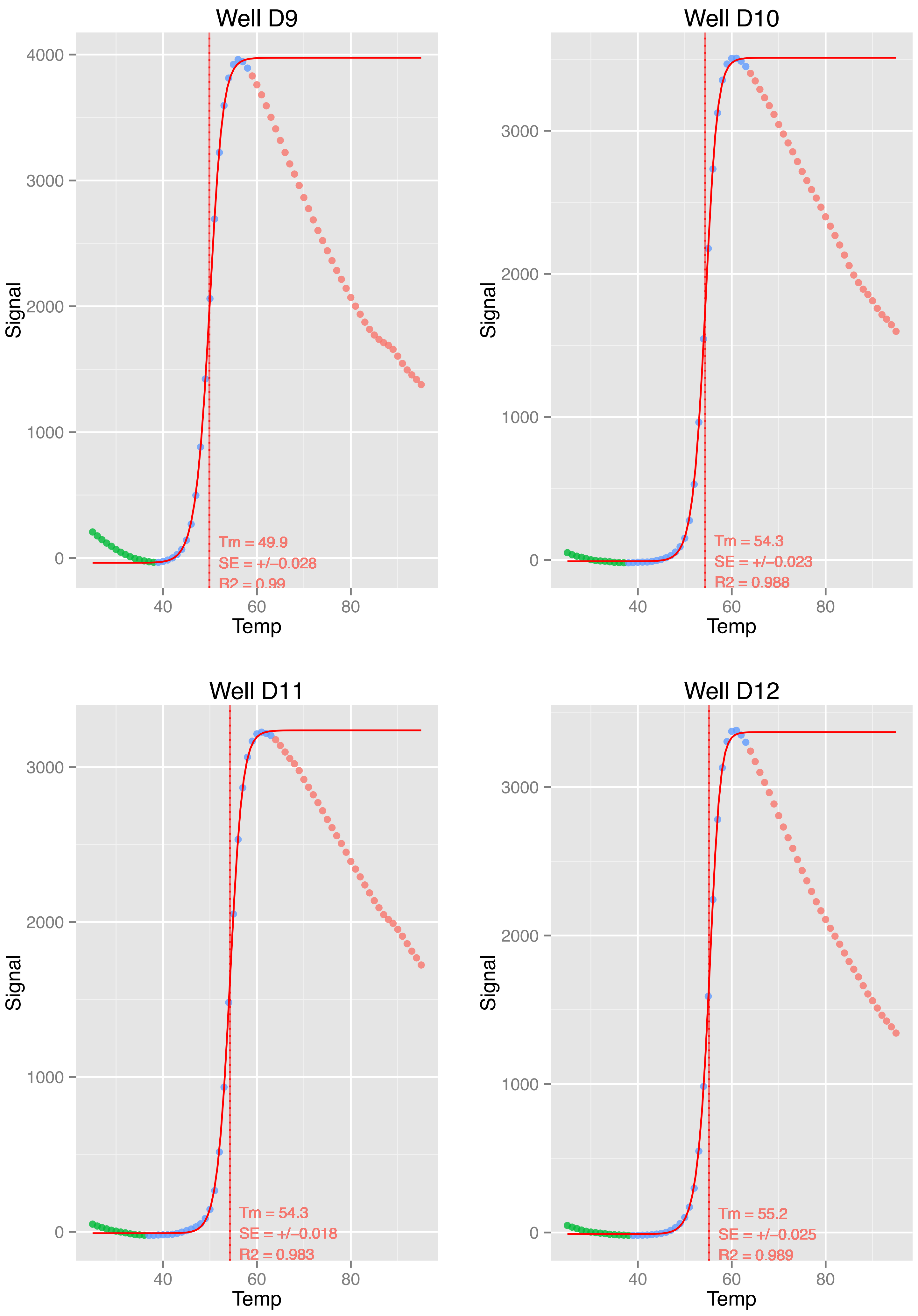

Well D9
4000
3000
2000
1000
0
Signal
40
60
80
Temp
Tm = 49.9
SE = +/−0.028
R2 = 0.99
Well D10
3000
2000
1000
0
Signal
40
60
80
Temp
Tm = 54.3
SE = +/−0.023
R2 = 0.988
Well D11
3000
2000
1000
0
Signal
40
60
80
Temp
Tm = 54.3
SE = +/−0.018
R2 = 0.983
Well D12
3000
2000
1000
0
Signal
40
60
80
Temp
Tm = 55.2
SE = +/−0.025
R2 = 0.989

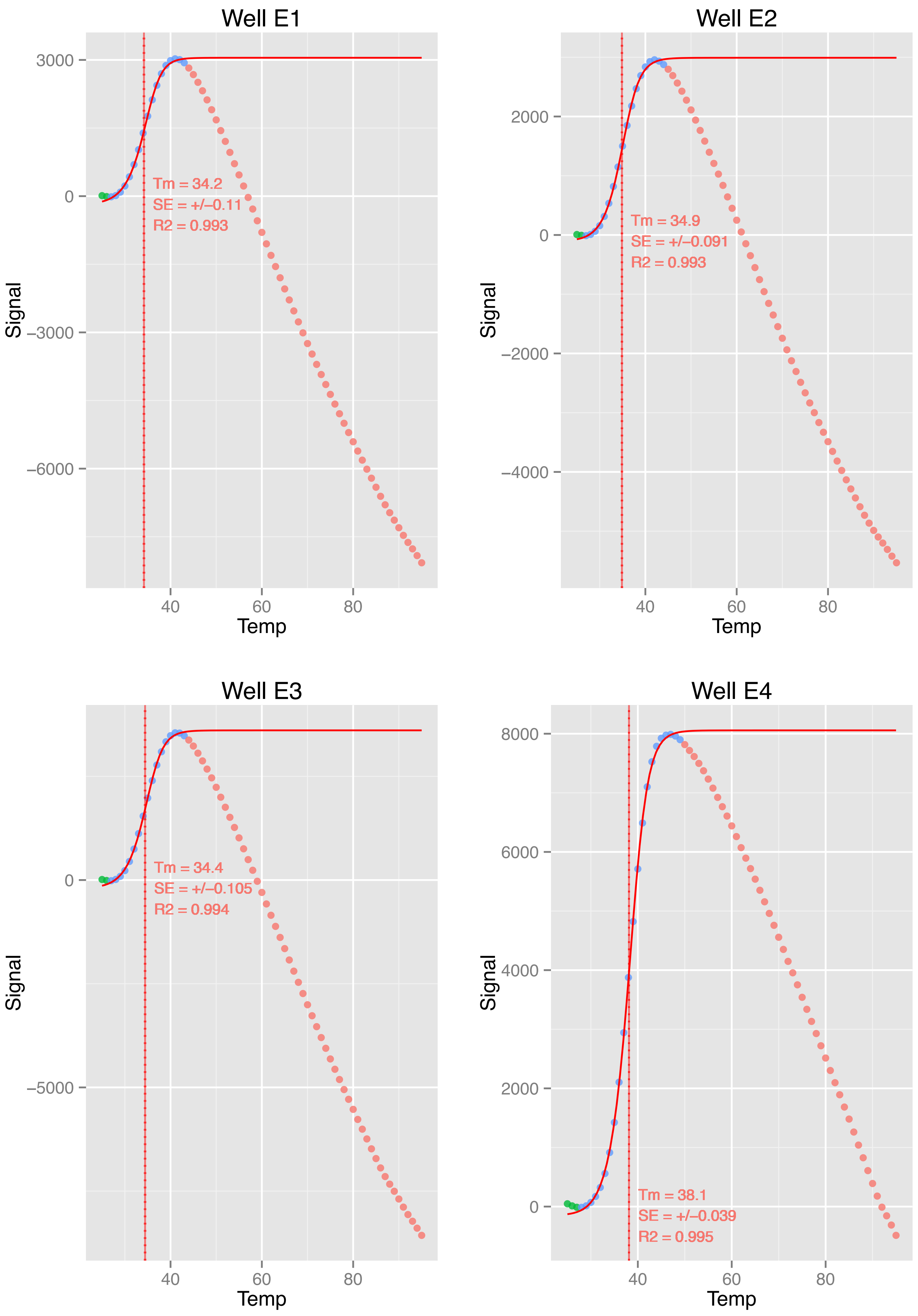
Well E1
3000
0
−3000
−6000
Signal
40
60
80
Temp
Tm = 34.2
SE = +/−0.11
R2 = 0.993
Well E2
2000
0
−2000
−4000
Signal
40
60
80
Temp
Tm = 34.9
SE = +/−0.091
R2 = 0.993
Well E3
0
−5000
Signal
40
60
80
Temp
Tm = 34.4
SE = +/−0.105
R2 = 0.994
Well E4
8000
6000
4000
2000
0
Signal
40
60
80
Temp
Tm = 38.1
SE = +/−0.039
R2 = 0.995

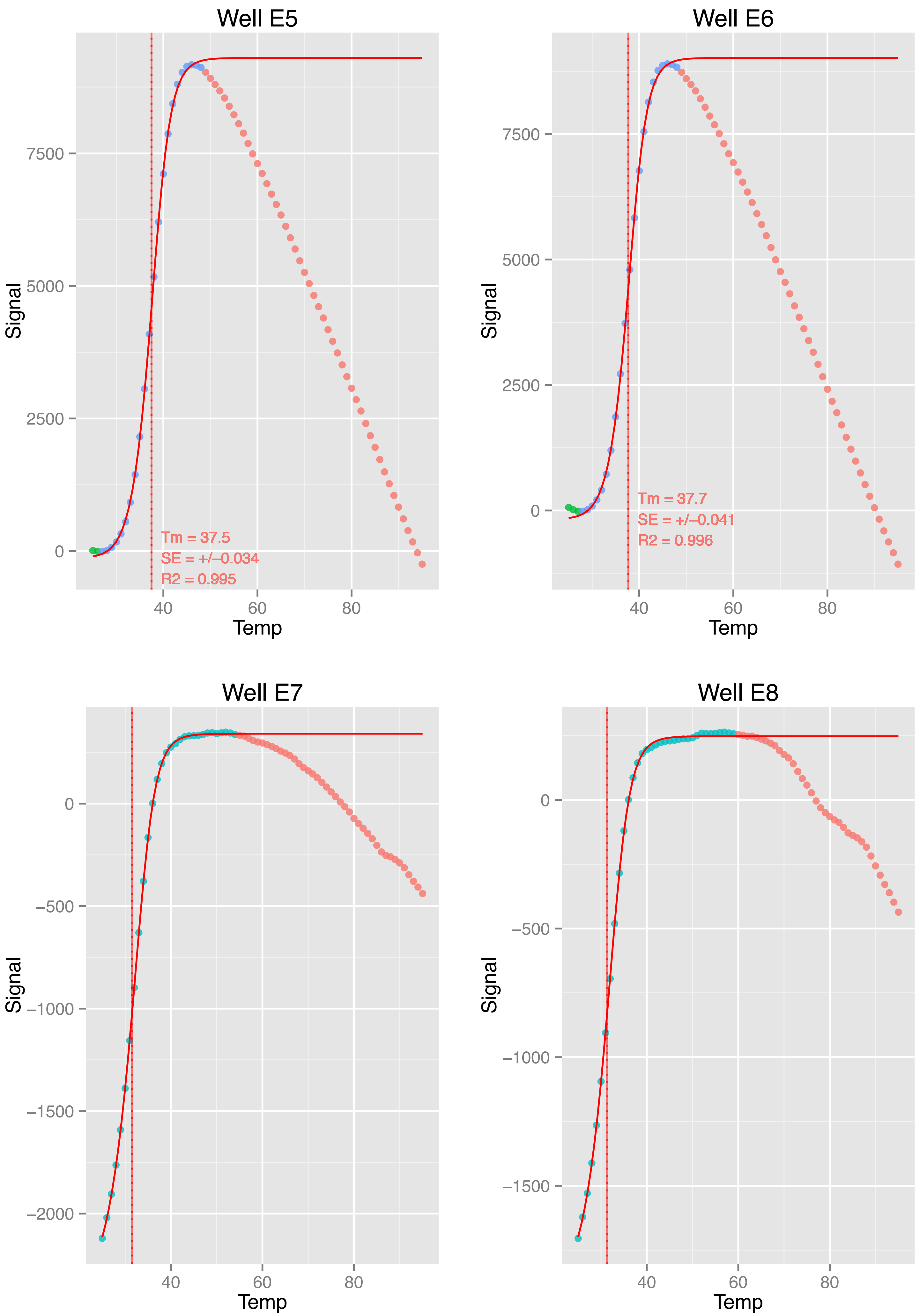

Well E5
Signal
Temp
Tm = 37.5
SE = +/−0.034
R2 = 0.995
Well E6
Signal
Temp
Tm = 37.7
SE = +/−0.041
R2 = 0.996
Well E7
Signal
Temp
Well E8
Signal
Temp

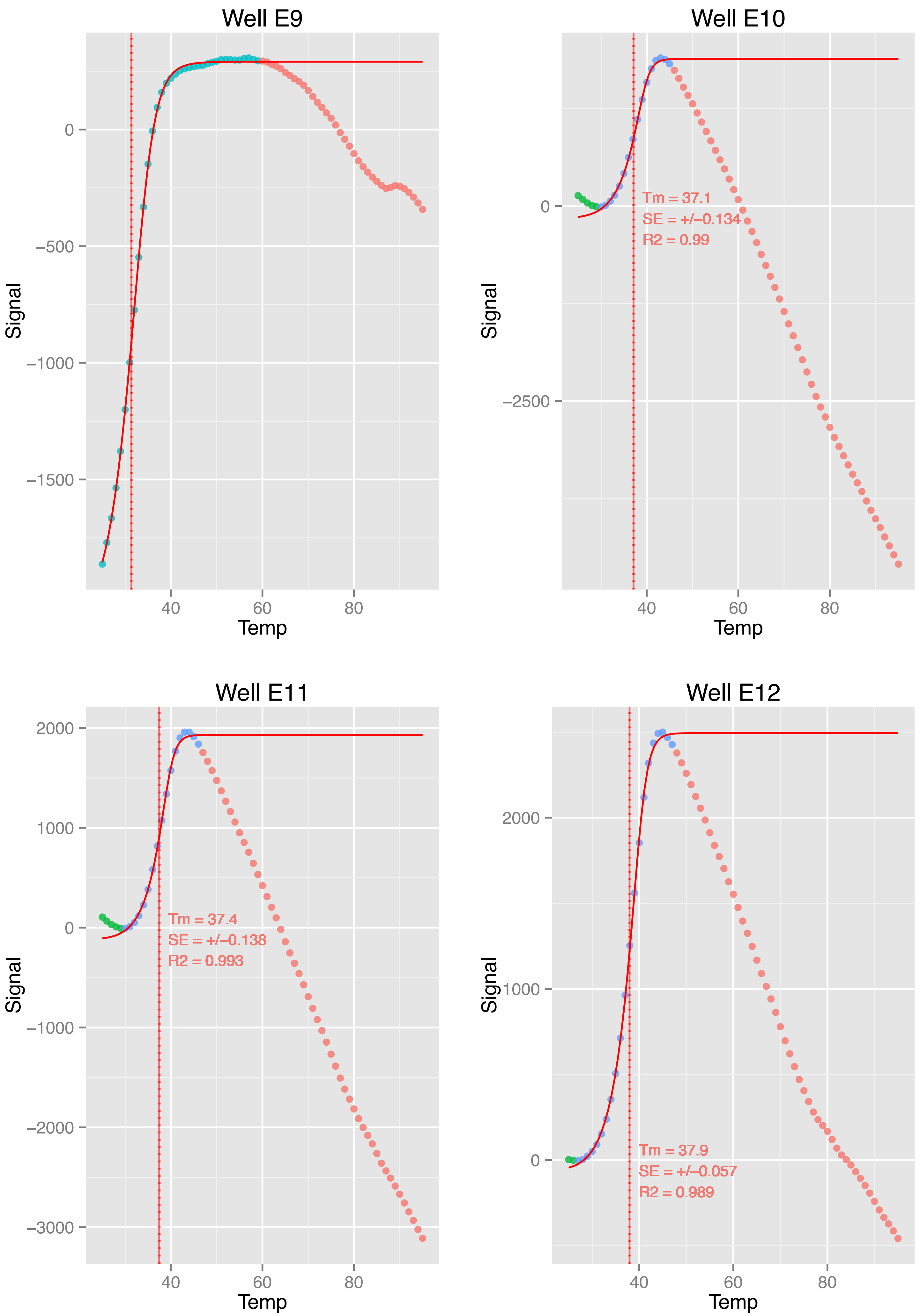
Well E9
Signal
0
−500
−1000
−1500
40
60
80
Temp
Well E10
Signal
0
−2500
Tm = 37.1
SE = +/−0.134
R2 = 0.99
40
60
80
Temp
Well E11
Signal
2000
1000
0
−1000
−2000
−3000
Tm = 37.4
SE = +/−0.138
R2 = 0.993
40
60
80
Temp
Well E12
Signal
2000
1000
0
Tm = 37.9
SE = +/−0.057
R2 = 0.989
40
60
80
Temp

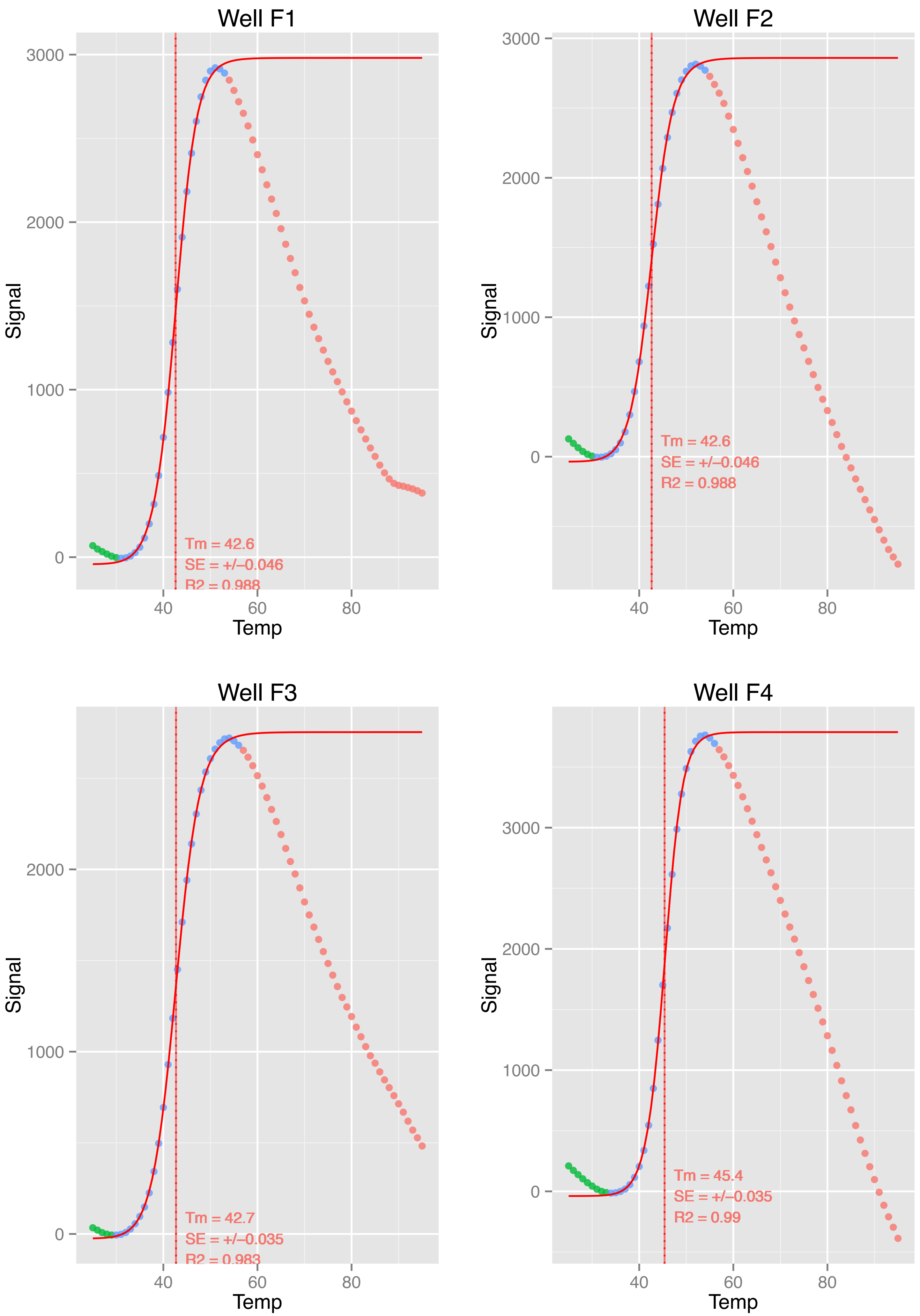
Well F1
Signal
Temp
Tm = 42.6
SE = +/–0.046
R2 = 0.988
Well F2
Signal
Temp
Tm = 42.6
SE = +/–0.046
R2 = 0.988
Well F3
Signal
Temp
Tm = 42.7
SE = +/–0.035
R2 = 0.983
Well F4
Signal
Temp
Tm = 45.4
SE = +/–0.035
R2 = 0.99

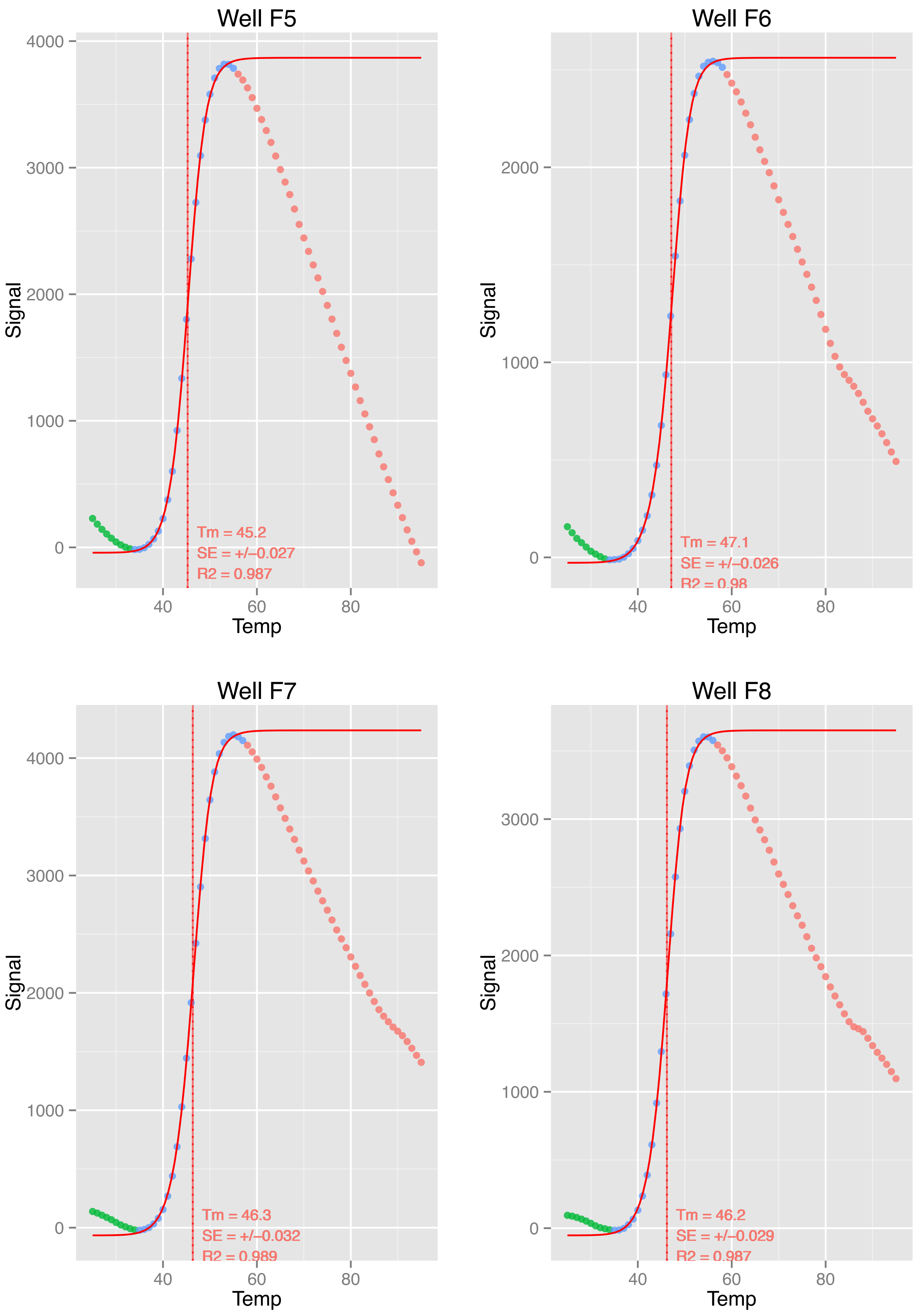
Well F5
Signal
Temp
Tm = 45.2
SE = +/−0.027
R2 = 0.987
Well F6
Signal
Temp
Tm = 47.1
SE = +/−0.026
R2 = 0.98
Well F7
Signal
Temp
Tm = 46.3
SE = +/−0.032
R2 = 0.989
Well F8
Signal
Temp
Tm = 46.2
SE = +/−0.029
R2 = 0.987

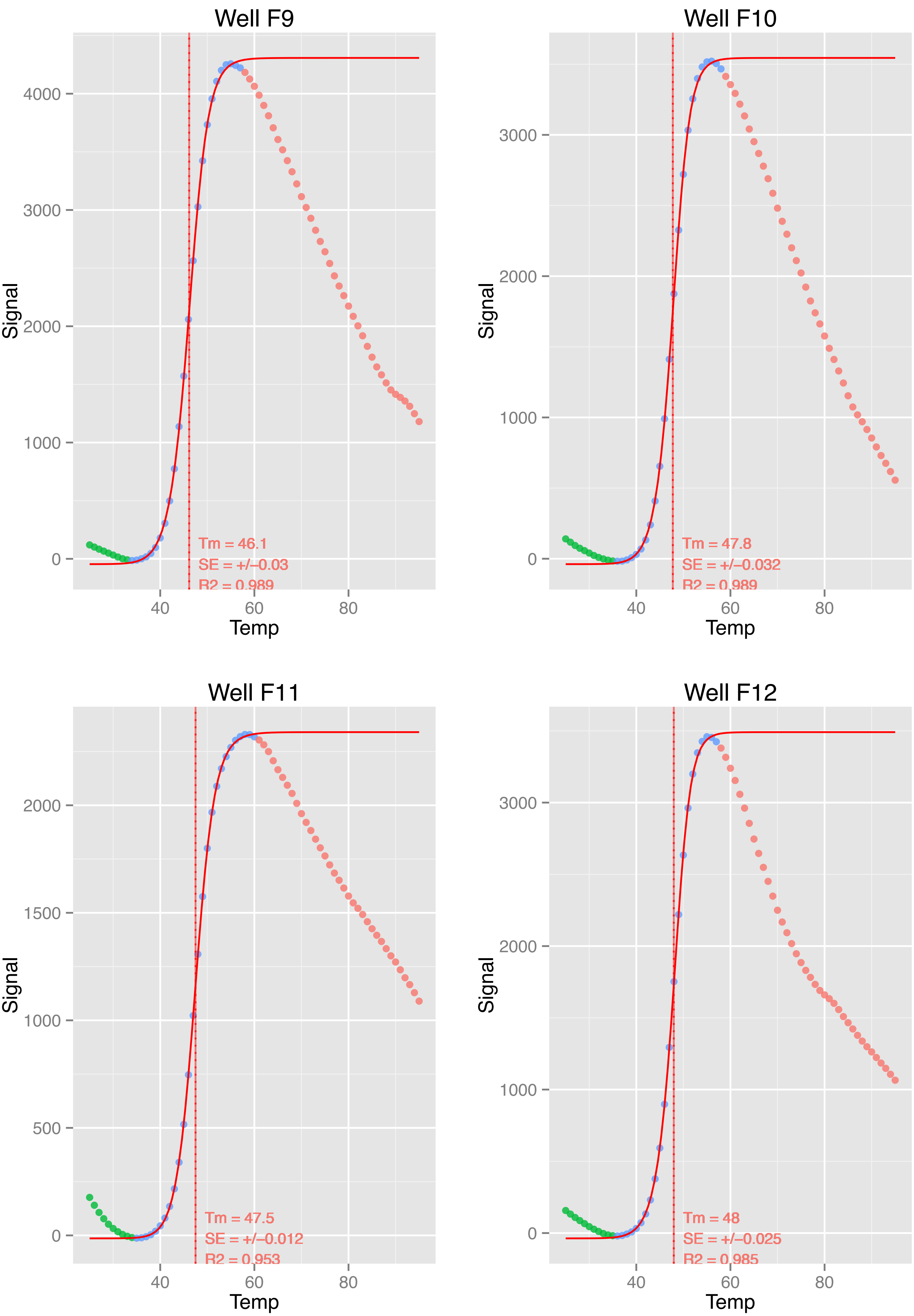
Well F9
Signal
Temp
Tm = 46.1
SE = +/−0.03
R2 = 0.989
Well F10
Signal
Temp
Tm = 47.8
SE = +/−0.032
R2 = 0.989
Well F11
Signal
Temp
Tm = 47.5
SE = +/−0.012
R2 = 0.953
Well F12
Signal
Temp
Tm = 48
SE = +/−0.025
R2 = 0.985
