# Supplementary material for: Mechanism of APTX nicked DNA sensing and pleiotropic inactivation in neurodegenerative disease
Source: EMBO J. 2018 Jun 22;37(14):e98875. doi: 10.15252/embj.201798875 (PMC6043908; doi:10.15252/embj.201798875)
Supplement: Supplementary file 3 — Movie EV1 [file EMBJ-37-e98875-s003.zip › Movie_EV1/Legend_Movie_EV1.rtf]

Movie EV1:Molecular morph depicting APTX engaging a nicked DNA substrate.  Substrate distortions are induced by APTX DNA binding.  In addition, DNA engagement remodels the APTX active site.
